# Anlotinib Inhibits PFKFB3-Driven Glycolysis in Myofibroblasts to Reverse Pulmonary Fibrosis

**DOI:** 10.3389/fphar.2021.744826

**Published:** 2021-09-16

**Authors:** Weimou Chen, Jinming Zhang, Wenshan Zhong, Yuanyuan Liu, Ye Lu, Zhaojin Zeng, Haohua Huang, Xuan Wan, Xiaojing Meng, Fei Zou, Shaoxi Cai, Hangming Dong

**Affiliations:** ^1^ Chronic Airways Diseases Laboratory, Department of Respiratory and Critical Care Medicine, Nanfang Hospital, Southern Medical University, Guangzhou, China; ^2^ Guangdong Provincial Key Laboratory of Tropical Disease Research, Department of Occupational Health and Medicine, School of Public Health, Southern Medical University, Guangzhou, China

**Keywords:** pulmonary fibrosis, anlotinib, glycolysis, PFKFB3, PCBP3

## Abstract

Idiopathic pulmonary fibrosis (IPF) is a fatal disease in which the normal alveolar network is gradually replaced by fibrotic scars. Current evidence suggests that metabolic alterations correlate with myofibroblast activation in IPF. Anlotinib has been proposed to have antifibrotic effects, but the efficacy and mechanisms of anlotinib against lung fibrosis have not been systematically evaluated. The antifibrotic effects of anlotinib were evaluated in bleomycin-induced mouse models and transforming growth factor-beta 1 (TGF-β1)-stimulated lung fibroblasts. We measured lactate levels, 2-NBDG glucose uptake and the extracellular acidification rate (ECAR) to assess glycolysis in fibroblasts. RNA-protein coimmunoprecipitation (RIP) and polysome analyses were performed to investigate novel mechanisms of glycolytic reprogramming in pulmonary fibrosis. We found that anlotinib diminished myofibroblast activation and inhibited the augmentation of glycolysis. Moreover, we show that PCBP3 posttranscriptionally increases PFKFB3 expression by promoting its translation during myofibroblast activation, thus promoting glycolysis in myofibroblasts. Regarding mechanism, anlotinib exerts potent antifibrotic effects by downregulating PCBP3, reducing PFKFB3 translation and inhibiting glycolysis in myofibroblasts. Furthermore, we observed that anlotinib had preventative and therapeutic antifibrotic effects on bleomycin-induced pulmonary fibrosis. Therefore, we identify PCBP3 as a protein involved in the regulation of glycolysis reprogramming and lung fibrogenesis and propose it as a therapeutic target for pulmonary fibrosis. Our data suggest that anlotinib has antifibrotic effects on the lungs, and we provide a novel mechanism for this effect. Anlotinib may constitute a novel and potent candidate for the treatment of pulmonary fibrosis.

## Introduction

Fibrosis can develop in most organs and cause organ failure. The most common type of lung fibrosis is idiopathic pulmonary fibrosis (IPF), which is highly prevalent and associated with a dramatically increased disease burden worldwide (Wynn and Ramalingam, 2012; Hutchinson et al., 2015). Overall, the development of new therapeutics should be pursued. Currently, only pirfenidone and nintedanib have been approved as therapeutics for IPF (Taniguchi et al., 2010; Sato et al., 2017), and as both drugs have limited efficacy (Spagnolo and Maher, 2017), there is an urgent need to identify new potential therapeutic agents for IPF patients.

Upon chronic microinjury to the alveolar epithelium, fibroblast activation and transdifferentiation into myofibroblasts are among the first responses detectable at the site of damage (Plantier et al., 2018). Myofibroblasts are characterized by *de novo* expression of α-smooth muscle actin (α-SMA), the formation of stress fibers, and enhanced abilities to proliferate, migrate, and produce extracellular matrix (ECM) (Hinz, 2012; Hinz et al., 2012; Liu et al., 2021). These cells drive a wound-healing response that relies on the deposition of collagen-rich ECM and activates transforming growth factor-beta 1 (TGF-β1) signaling (Kenyon et al., 2003; Sapudom et al., 2015; Nigdelioglu et al., 2016). This transient response must be tightly controlled, otherwise it can become persistent and lead to excessive matrix accumulation and fibrosis. Understanding the molecular bases of fibroblast activation is therefore essential in identifying novel and efficient antifibrotic therapeutic targets to reduce the incidence, morbidity and mortality of people suffering from clinically refractory disorders, including IPF.

Metabolic perturbation is implicated in the pathogenesis of several kinds of tissue fibrosis (DeBerardinis and Thompson, 2012; Chen et al., 2018), including pulmonary fibrosis (Para et al., 2019; Bueno et al., 2020). To cope with the high energy demands of myofibroblasts, including increases in proliferation and matrix production, it is reasonable that activated myofibroblasts exhibit augmented aerobic glycolysis to meet additional bioenergetic and biosynthetic demands, even in oxygen-rich conditions, similar to observations in many cancer cells and other nonmalignant proliferating cells. A major driver of glycolysis is 6-phosphofructo-2-kinase/fructose-2,6-bisphosphatase-3 (PFKFB3), which produces fructose-2,6-bisphosphate (F2,6BP), the most potent allosteric activator of the glycolytic rate-limiting enzyme phosphofructokinase-1 (PFK1) (Van Schaftingen et al., 1982; Cao et al., 2019). Previous studies have shown that augmentation of aerobic glycolysis is an essential step during myofibroblast activation (Para et al., 2019). Ramping down glycolysis is effective in diminishing myofibroblast activation, thus limiting lung fibrosis. However, metabolism-based therapeutics for treating fibrotic disorders are still lacking.

Anlotinib (AL3818) hydrochloride is a novel multitargeted tyrosine kinase inhibitor (TKI) that targets the receptor tyrosine kinases vascular endothelial growth factor receptor (VEGFR) 1 thru 3, epidermal growth factor receptor (EGFR), fibroblast growth factor receptor (FGFR) 1 thru 4, platelet-derived growth factor receptor (PDGFR) α and β, and stem cell factor receptor (Sun et al., 2016; Xie et al., 2018). Many studies have reported the therapeutic effects of anlotinib in several diseases, such as nonsmall cell lung cancer (Liang et al., 2019), endometrial cancers (Taurin et al., 2018) and osteosarcoma (Liang et al., 2019). Interestingly, the targets of anlotinib are similar to those of nintedanib, a drug that has been approved for the treatment of IPF. A recent report suggested that intraperitoneal administration of anlotinib attenuates bleomycin-induced lung fibrosis in mice by suppressing the TGF-β signaling pathway (Ruan et al., 2020). Nevertheless, the mechanism by which anlotinib resolves lung fibrosis and whether anlotinib may be therapeutically used to improve lung function are not well understood.

In the current study, we analyzed the antifibrotic effect of anlotinib on TGF-β1-induced fibroblast transdifferentiation and bleomycin-induced pulmonary fibrosis. Our data suggest that anlotinib therapy decreases fibrotic markers *in vitro* and *in vivo*. The antifibrotic effect of anlotinib is associated with inhibition of PFKFB3-dependent glycolysis, which is posttranscriptionally regulated by the RNA binding protein PCBP3. These findings provide a theoretical basis for the clinical development and application of anlotinib for the treatment of pulmonary fibrosis.

## Materials and Methods

### Isolation of Primary Mouse Fibroblast Cultures

Normal mouse primary fibroblasts were generated by culturing the lungs of C57BL/6 as previously described (Meng et al., 2014). The cells were cultured in Dulbecco’s modified Eagle’s medium (DMEM, Gibco, United States) supplemented with 15% fetal bovine serum (FBS, PAN, German). The cells were cultured at 37°C in 5% CO2 and 95% humidity. Unless specifically noted, all experiments were performed with cells at passage 3.

### Cell Lines

Human lung fibroblast line IMR90 was purchased from American Type Culture Collection (Manassas, VA). IMR90 were maintained in DMEM supplemented with 10% FBS, 100 units/mL penicillin, and 100 g/ml streptomycin in 5% CO2 and 95% humidity at 37°C.

### Western Blotting

Cells or dissected mouse lung tissue samples were lysed in ice-cold RIPA lysis buffer with protease inhibitors. Protein concentrations were determined using a BCA Protein Quantitative Analysis Kit (Fudebio-tech) after which protein samples were separated by 8–12% SDS-PAGE and transferred onto polyvinylidene difluoride membranes (Millipore). The membranes were then incubated at room temperature for 1 h in TBST containing 5% BSA. After blocking, the membranes were incubated with primary antibodies for 24 h at 4°C.The following primary antibodies were used: anti-Fibronectin (Abcam, ab268020); anti-Collagen I (affinity, AF7001); anti-alpha smooth muscle (Abcam, ab5694); anti-PFKFB3 (Abcam, ab181861); anti-Beta actin (proteintech, 66009-1-Ig); anti-Hexokinase 2 (proteintech, 22029-1-AP); anti-PKM2 (Proteintech, 15822-1-AP); anti-LDHA (Proteintech, 19987-1-AP); anti-LDHB (Proteintech, 14824-1-AP); and anti-PCBP3 (Abcam, ab154252). Then, the membranes were washed three times with TBST and incubated with donkey anti-rabbit IgG H&L (Abcam, ab175772) for 1 h at room temperature. The membranes were developed using the ECL method according to the manufacturer’s instructions (Millipore) and detected on a GeneGnome XRQ chemiluminescence imaging system (Syngene). ImageJ was used to calculate the relative density of proteins.

### Immunofluorescence Staining

The culture medium was washed away with PBS. The cultured cells were fixed with 4% paraformaldehyde for 30 min. Then, the samples were permeabilized with 0.5% Triton X-100 in PBS for 10 min, blocked with 1% BSA in PBS for 1 h at room temperature, and incubated with primary antibodies at 4°C overnight. The primary antibodies included anti- Fibronectin (Abcam, ab268020), anti-alpha smooth muscle (Abcam, ab5694) and anti-PCBP3 (Abcam, ab154252). Then, the cells were washed three times with PBS and incubated with goat anti-rabbit IgG/Alexa Fluor 555-conjugated secondary antibodies (Biosynthesis, bs-0296GA488 and bs-0295G-AF555) for 1 h at room temperature followed by 10 min of DAPI (4’,6-diamidino-2-phenylindole dihydrochloride) staining to visualize cell nuclei visualization as previously described (Chen et al., 2021).

### Quantitative RT-PCR (qPCR)

Total RNA was isolated from primary mouse lung fibroblasts using RNA MiniPrep Kits (Zymo Research, R2050). Reverse transcription reactions were performed with a PrimeScriptTM II 1st strand cDNA synthesis Kit (Takara, 6210A/B) according to the manufacturer’s recommendations. qPCR analysis was performed using a HiScript RT- SuperMix for qPCR kit (Vazyme, R223-01) with a CFX96 Touch Real-Time PCR Detection System. The mRNA levels of target genes were normalized to the β-actin mRNA level. Primers used for qPCR are listed in (Table 1).

**TABLE 1:** List of primer sequences used in this study.

Gene	Species	Forward primer	Reverse primer
β-actin	Mus musculus	GGC​TGT​ATT​CCC​CTC​CAT​CG	CCA​GTT​GGT​AAC​AAT​GCC​ATG​T
PFKFB3	Mus musculus	CCC​AGA​GCC​GGG​TAC​AGA​A	GGG​GAG​TTG​GTC​AGC​TTC​G

### Wound-Healing Migration Assay

Cells were seeded in six-well plates and grown until they reach 100% confluence. A “wound” was subsequently created with a sterile 100 μL pipette tip. The cells were pretreated with anlotinib (1 µM) for 3 h and then exposed to TGF-β1 (10 ng/ml) for an additional 24 h. After 24 h, the cells were fixed with 4% paraformaldehyde, and images were obtained using a fluorescence microscope. Wound area can be calculated by manually tracing the cell-free area in captured images using the ImageJ public domain software (NIH, Bethesda, MD).

### Cell Proliferation Assay

Cell proliferation was determined by the CCK-8 Kit (Dojindo Laboratories) according to the manufacturer’s instructions. Briefly, 10 μL of CCK-8 solution was added to cultured cells in each well, followed by incubation at 37°C for 1 h. The OD values were measured at 450 nm using a microplate reader. EdU staining was conducted using the BeyoClick™ EdU Cell Proliferation Kit with Alexa Fluor 594 (Beyotime, Cat. No: C00788L). Cells were washed with PBS. Fresh DMEM was added, and then, 10 µM EdU was added into the medium. The cells were incubated for 2 h at 37°C/5% CO2. After the incubation, the cells were washed with PBS to remove the DMEM and the free EdU probe. The cells were then fixed in 4% paraformaldehyde at room temperature for 30 min before being stained with DAPI for 3 min. After an additional wash in PBS, the cells were observed under Nikon ECLIPSE TS100 (Japan).

### Glucose Uptake Assay

Primary mouse lung fibroblasts were pretreated with anlotinib (1 µM) for 3 h and then exposed to TGF-β1 (10 ng/ml) for an additional 24 h. Then, the four types of cells were detached and transferred to a 96-well plate in fresh growth medium at a density of 10,000 cells per well for the direct 2-NBDG glucose uptake assay. The cells were rinsed twice with PBS. Glucose uptake was initiated by the addition of 100 μM 2-NBDG to each well. After 30 min, the medium was removed. The plates were then rinsed with PBS, and the fluorescence was measured at an excitation wavelength of 485 nm and an emission wavelength of 535 nm.

### Intracellular and Extracellular Lactate Analysis

To measure lactate production, cells were treated as described for the glucose uptake assay. One hundred thousand cells were then plated into a 12-well plate and incubated in DMEM containing 10% FBS for 10 h. To measure the secretion of lactate, the media were removed, and the cells were incubated in FBS-free DMEM. After incubation for 1 h, the supernatant was collected to measure lactate production (Biovision). The reaction mixture was incubated for 30 min at room temperature in the dark. The lactate levels were measured at 450 nm in a microplate reader and normalized to the protein concentrations. To measure the lactate levels in mouse lung tissue, 10 mg of lung tissues was isolated and homogenized in assay buffer (Biovision). The samples were centrifuged, and the soluble fractions were measured and normalized to the protein concentrations.

### Extracellular Acidification Rate

The extracellular acidification rate (ECAR) was measured using the Agilent Seahorse XFp Extracellular Flux Analyzer (Seahorse Bioscience). Experiments were performed according to the manufacturer’s instructions. ECAR were measured using Seahorse XF Glycolysis Stress Test Kit (Agilent Technologies). Briefly, cells were transfected or infected as in glucose uptake assay. The transfected cells were harvested and the cell number was counted. After baseline measurements, glucose, the oxidative phosphorylation inhibitor oligomycin, and the glycolytic inhibitor 2-DG were sequentially injected into each well at the indicated time points. Data were analysed by Seahorse XFp Wave software. ECAR is reported in mpH/minute. The cells in each well were digested by trypsin digestion (Gibco, United States), and count cell numbers by cell counting chamber. The results were normalized to normalized to cell number in each well.

### RNA Immunoprecipitation (RIP)

Cells were rinsed twice with ice-cold PBS and lysed with an equal pellet volume of RIPA-2 buffer. Protein-A Dynabeads (Invitrogen) were incubated with either mouse IgG or FLAG antibody (Abcam, ab205606). Beads coated in antibody were resuspended in NT2 buffer. Thawed and clarified lysates were added and the bead/antibody/lysate mixture was incubated at 4°C overnight rotating end-over-end. Beads were washed with cold NT2 buffer five times. Proteinase K treatment released RNAs from bound proteins and input and bound RNA was isolated with TRIzol (Invitrogen) and reverse transcribed as described above.

### Polysome Analysis

Cells were transfected with empty vector or Flag-PCBP3 and incubated with 100 g/ml cycloheximide for 10 min and lysed with polysome extraction buffer containing 20 mM Tris–HCl, pH 7.5, 100 mM KCl, 5 mM MgCl2 and 0.5% NP-40 as previously described (Kim et al., 2015). Cytoplasmic lysates were fractionated by ultracentrifugation through 10–50% linear sucrose gradients and divided into 12 fractions. The total RNA in each fraction was extracted and analyzed by quantitative RT-PCR analysis.

### Overexpression Experiments and RNA Interference

The Plasmid vector encoding PCBP3 and the empty vector were purchased from Hanbio (Shanghai, China). Primary mouse lung fibroblasts were cultured in six well plates (105 cells/well) and added with 2.5 μg of target plasmid per well. After 12 h, the transfection medium was changed to normal medium. Effects of overexpression on mRNA and protein levels were examined 36 h later. The siRNA targeting mouse PFKFB3 (PFKFB3 siRNA: 5′- CCU​CUU​GAC​CCU​GAU​AAA​UTT-3′) were synthesized by Genepharma Co. (Shanghai, China). Primary mouse lung fibroblasts were cultured in six well plates (105 cells/well) and transfected using Lipofectamine 3,000 (Invitrogen, CA) with PFKFB3 siRNA or negative control siRNA (NC siRNA) for 48 h following the manufacturer’s instructions.

### Animal Experiments

All experiments were conducted in accordance with protocols approved by the Southern Medical University Institutional Animal Care and Use Committee. Female mice (C57BL/6), 6–8 weeks of age, were purchased from Southern Medical University. The mice were kept on a 12 h light-dark cycle with free access to food and water. For bleomycin administration, the mice were anesthetized with 2, 2, 2-tribromoethanol (Sigma-Aldrich), followed by intratracheal instillation of BLM (5 U/kg, i. t.) in 50 μL phosphate-buffered saline (PBS) or equally volume PBS for 21 days. The mice were administered dimethyl sulfoxide (DMSO) (control group) or anlotinib (1 mg/kg, i. p.) once daily for 21 consecutive days. Further experiments were designed to measure the effects of delayed anlotinib administration. Anlotinib treatment was initiated 1 week after exposure to bleomycin, and the mice were administered with anlotinib (1 or 2 mg/kg/day) for 2 weeks, and the mice were sacrificed at day 21. The lungs were harvested for further analyses.

### Pulmonary Function Test

At endpoint, at least 5 mice from each group were anesthetized with 2,2,2-tribromoethanol in saline, tracheotomized below the larynx, and intubated with a tracheal cannula. After the surgery, the mice were placed inside the plethysmographic chamber and the cannula was connected to the machine. Pulmonary function was measured by pulmonary function test system (BUXCO, United States). The system’s software automatically records and displays the pulmonary function parameters.

### Hydroxyproline Assay

Lung collagen content was measured with a hydroxyproline (HYP) kit (Nanjing Jian Cheng Institute, Nanjing, China). The lung tissues were prepared for hydrolysis, adjusting the PH value to 6.0–6.8. Subsequently, the developing solution was added to the tissues that were incubated at 37°C for 5 min. Absorbance was read at 550 nm using a microplate reader. Data were expressed as micrograms (µg) of HYP per mg of wet lung tissue.

### Materials

TGF-β1 were purchased from R&D Systems, Inc. (Minneapolis, MN, United States). Anlotinib dihydrochloride (AL3818, S8726) were purchased from Selleck (Houston, TX, United States).

### Statistical Analysis

The results are expressed as the means ± standard deviation (SD). Multigroup comparisons were performed using one-way ANOVA. Student’s t-test was used for comparisons between two groups. A *p* value of less than 0.05 was considered significant. Replicates consisted of at least three independent experiments. Analyses were performed on SPSS version 25.0 (IBM) for Windows and GraphPad Prism version 6.0 (GraphPad Software, CA).

## Results

### Anlotinib Represses Myofibroblast Activation and the Profibrogenic Phenotype *in vitro*

**FIGURE 1:**
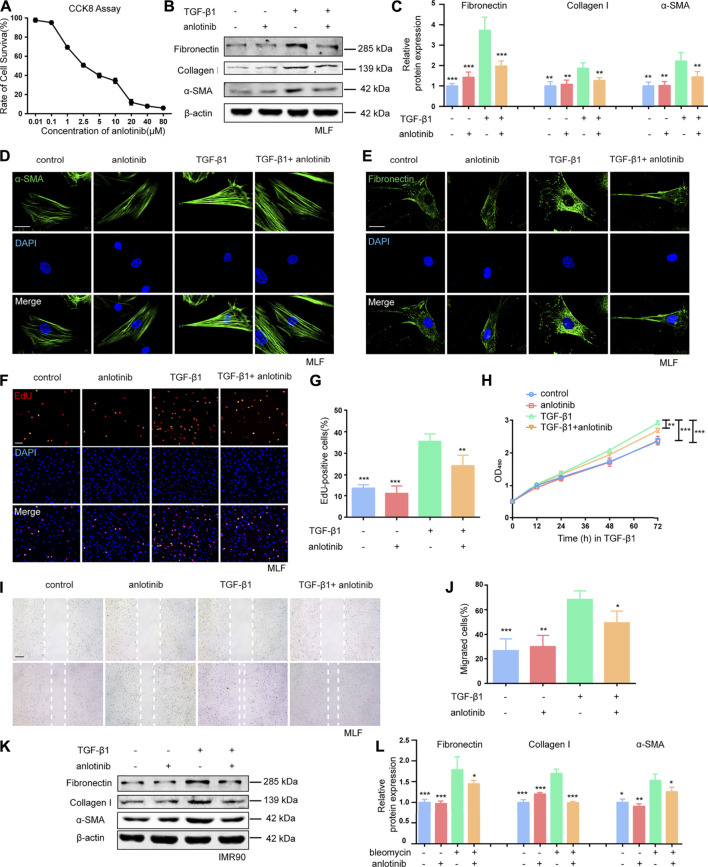
Anlotinib represses myofibroblast activation and the profibrogenic phenotype *in vitro*.**(A)** Dose-dependent cytotoxicity of anlotinib in primary mouse lung fibroblast isolated from healthy mice by CCK-8. **(B)** Western blots analysis of Fibronectin, Collagen I, α-SMA and β-actin in mouse lung fibroblasts treated with anlotinib (1 µM) for 3 h and then exposed to TGF-β1 (10 ng/ml) for 3  h, followed by TGF-β1 for an additional 24 h. **(C)** Quantification for the indicated protein (mean ± SD, n = 3). Immunofluorescence for α-SMA (green) **(D)** and Fibronectin (green) **(E)**. DAPI-stained nuclei (blue). Scale bar, 25 µm. **(F)** An EdU assay was used to observe the proliferative cells. Scale bar, 100 µm. **(G)** The number of EdU-positive cells was recorded (mean ± SD, n = 3). **(H)** A cck8 assay was used to observe the proliferative cells. **(I)** Representative images to show scratch-wound assay. Scale bars, 100 µm. Experiments were performed as in B. Images were taken 0, and 24 h after assay (white lines indicate wound edge). **(J)** Quantitative analysis of migration distance (mean ± SD, n = 3). **(K)** Western blots analysis of Fibronectin, Collagen I, α-SMA and β-actin in IMR90 cells treated with anlotinib (1 µM) for 3 h and then exposed to TGF-β1 (10 ng/ml) for an additional 24 h. **(L)** Quantification for the indicated proteins in IMR90 cells (mean ± SD, n = 3). *p < 0.05, **p < 0.01, ***p < 0.001 VS TGF-β1-treated group (ANOVA).

Given that TGF-β1 is the predominant cytokine that stimulates the differentiation of lung fibroblasts into myofibroblasts and induces ECM production (Sapudom et al., 2015; Huang et al., 2020), we examined the effect of anlotinib (the chemical structure is shown in Supplementary Figure S1) on TGF-β1-induced activation of primary mouse lung fibroblasts (MLFs). The CCK-8 assay results showed that anlotinib did not cause significant cytotoxicity at doses of 1 µM (Figure 1A). To mimic the inhibitory effect of anlotinib on the progression of lung fibrosis, primary MLFs were pretreated with anlotinib (1 µM) for 3 h and then exposed to TGF-β1 (10 ng/ml) for an additional 24 h. Our results demonstrated that TGF-β1 induced the expression of fibronectin, collagen I, and α-SMA, but anlotinib reversed the expression of these fibrotic markers (Figures 1B,C). Immunofluorescence analysis of α-SMA and fibronectin showed similar results (Figures 1D,E). We also examined whether anlotinib affected the proliferation and migration of fibroblasts, which have been shown to significantly contribute to many fibrotic pathologies (Jarman et al., 2014; Huang et al., 2020). As shown by the EdU (Figures 1F,G) and CCK-8 results (Figure 1H), anlotinib treatment prevented the TGF-β1-induced proliferation of primary MLFs. Moreover, anlotinib inhibited the TGF-β1-induced migration of fibroblasts (Figures 1I,J). These results were confirmed in the human IMR90 cell line (Figures 1K, L and Supplementary Figures S2A–D). These data indicate that anlotinib can repress myofibroblast activation and the profibrogenic phenotype *in vitro*.

### Anlotinib Inhibits PFKFB3-Driven Glycolysis in Lung Myofibroblasts

**FIGURE 2:**
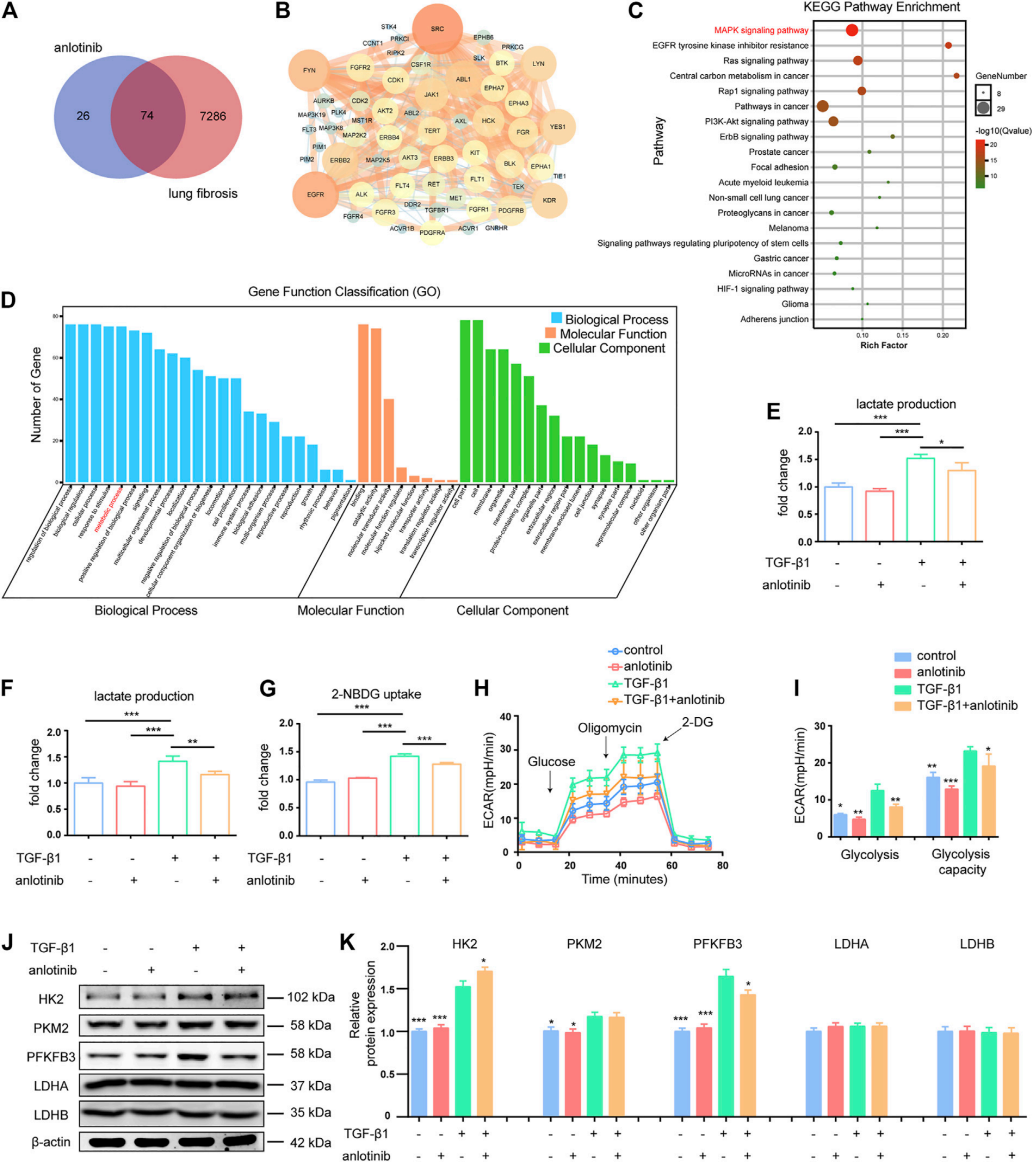
Anlotinib inhibits PFKFB3-driven glycolysis in lung myofibroblasts. **(A)** Venn diagram to show the overlaps between anlotinib targets and lung fibrosis-related targets. **(B)** Protein-protein interaction (PPI) network of common targets between anlotinib and IPF. **(C)** The KEGG enrichment analysis of 74 targets of common targets. **(D)**The GO enrichment for each section listed. The mouse lung fibroblasts were pretreated with anlotinib (1 µM) for 3 h and then exposed to TGF-β1 (10 ng/ml) for an additional 24 h, and then the cells were lysed and lactate contents in the cellular lysates **(E)** and culture media **(F)** were determined. The data are presented as fold change relative to the levels of the untreated control group (mean ± SD, n = 3). **(G)** Glucose uptake detected with 2-NBDG were determined. The data are presented as fold change relative to the levels of the untreated control group (mean ± SD, n = 3). **(H)** Extracellular acidification rate (ECAR) was assessed. **(I)** Glycolysis and glycolysis capacity were quantified and shown as histograms (mean ± SD, n = 3). **(J)** Western blot analysis of HK2、PKM2、PFKFB3、LDHA and LDHB, β-actin was used as a loading control. **(K)** Quantification of HK2、PKM2、PFKFB3、LDHA and LDHB protein levels relative to β-Actin is shown (mean ± SD, n = 3). I, K, *p < 0.05, **p < 0.01, ***p < 0.001 VS TGF-β1-treated group by ANOVA.

To investigate the potential antifibrotic mechanisms of anlotinib, we estimated the most likely macromolecular targets of anlotinib and obtained 100 potential targets through SwissTargetPrediction (Table 2) (Gfeller et al., 2014). A total of 7,360 lung fibrosis-related targets were obtained from the GeneCards database (Table 3) (Safran et al., 2002). To clarify the interaction between potential anlotinib targets and lung fibrosis-related targets, the intersection of the targets was mapped by drawing a Venn diagram and constructing a target network (Figure 2A). Seventy-four common targets were shared between the potential anlotinib targets and lung fibrosis-related targets (Table 4). STRING (version 11.0) was used for protein-protein interaction (PPI) analysis (Figure 2B) (Szklarczyk et al., 2019). Bioinformatics analysis data identified mitogen-activated protein kinase (MAPK) signaling pathway-related genes as the top hits among the 74 genes (Figure 2C). Given that the MAPK pathway is well recognized as a metabolic regulator and that many of these genes control metabolic processes (Figure 2D) (Ho et al., 2004; Papa et al., 2019; Hu et al., 2020; Wang F. et al., 2020), we first confirmed the presence of glycolytic alterations in lung myofibroblasts. We directly measured the levels of lactate and found that both intracellular and extracellular lactate levels in lung myofibroblasts treated with TGF-β1 were significantly increased (Figures 2E,F). Consistent with the augmented glycolysis in lung myofibroblasts, these cells also demonstrated increased glucose consumption (Figure 2G). However, anlotinib treatment decreased the production and secretion of lactate and reduced the consumption of glucose (Figures 2E–G). Accordingly, extracellular acidification rate (ECAR) analysis indicated that treatment with TGF-β1 increased glycolysis and glycolytic activity in primary MLFs, both of which were also reduced by anlotinib (Figures 2H,I). To delineate the mechanisms by which anlotinib inhibits the augmented glycolysis observed in lung myofibroblasts, we assessed the expression of key glycolytic enzymes in these cells. We found that PFKFB3 was induced by TGF-β1 in lung fibroblasts and that anlotinib significantly decreased its expression at the protein level (Figures 2J,K). PFKFB3 is not a rate-limiting glycolytic enzyme; instead, PFKFB3 catalyzes the conversion of fructose-6-phosphate to fructose-2,6-bisphosphate, which is an allosteric activator of PFK1 and a potent stimulator of glycolysis (Atsumi et al., 2002; De Bock et al., 2013). Taken together, these data suggest that anlotinib can abrogate the PFKFB3-driven increase in glycolysis, participating in myofibroblast activation.

**TABLE 2:** Targets of anlotinib obtained through SwissTargetPrediction.

Target	Common name	Uniprot ID	ChEMBL ID	Target class	Probability*	Known actives (3D/2D)
Tyrosine-protein kinase receptor UFO	AXL	P30530	CHEMBL4895	Kinase	0.106165761464	72/8
Tyrosine-protein kinase receptor TYRO3	TYRO3	Q06418	CHEMBL5314	Kinase	0.106165761464	53/3
Proto-oncogene tyrosine-protein kinase MER	MERTK	Q12866	CHEMBL5331	Kinase	0.106165761464	46/3
Receptor protein-tyrosine kinase erbB-2	ERBB2	P04626	CHEMBL1824	Kinase	0.106165761464	178/5
Tyrosine-protein kinase BRK	PTK6	Q13882	CHEMBL4601	Kinase	0.106165761464	22/2
Serine/threonine-protein kinase Aurora-B	AURKB	Q96GD4	CHEMBL2185	Kinase	0.106165761464	143/14
Vascular endothelial growth factor receptor 1	FLT1	P17948	CHEMBL1868	Kinase	0.106165761464	79/18
Epidermal growth factor receptor erbB1	EGFR	P00533	CHEMBL203	Kinase	0.106165761464	597/31
Vascular endothelial growth factor receptor 2	KDR	P35968	CHEMBL279	Kinase	0.106165761464	485/95
Tyrosine-protein kinase SRC	SRC	P12931	CHEMBL267	Kinase	0.106165761464	448/42
Hepatocyte growth factor receptor	MET	P08581	CHEMBL3717	Kinase	0.106165761464	314/108
Serine/threonine-protein kinase GAK	GAK	O14976	CHEMBL4355	Kinase	0.106165761464	16/2
Kinesin-1 heavy chain/Tyrosine-protein kinase receptor RET	RET	P07949	CHEMBL2041	Kinase	0.106165761464	72/11
ALK tyrosine kinase receptor	ALK	Q9UM73	CHEMBL4247	Kinase	0.106165761464	207/3
Tyrosine-protein kinase ABL	ABL1	P00519	CHEMBL1862	Kinase	0.106165761464	127/11
Stem cell growth factor receptor	KIT	P10721	CHEMBL1936	Kinase	0.106165761464	106/10
Activin receptor type-1	ACVR1	Q04771	CHEMBL5903	Kinase	0.106165761464	29/1
Vascular endothelial growth factor receptor 3	FLT4	P35916	CHEMBL1955	Kinase	0.106165761464	37/8
Tyrosine-protein kinase receptor FLT3	FLT3	P36888	CHEMBL1974	Kinase	0.106165761464	156/14
Platelet-derived growth factor receptor alpha	PDGFRA	P16234	CHEMBL2007	Kinase	0.106165761464	57/14
Fibroblast growth factor receptor 1	FGFR1	P11362	CHEMBL3650	Kinase	0.106165761464	189/9
TGF-beta receptor type I	TGFBR1	P36897	CHEMBL4439	Kinase	0.106165761464	78/4
Tyrosine-protein kinase LCK	LCK	P06239	CHEMBL258	Kinase	0.106165761464	167/19
Tyrosine-protein kinase BTK	BTK	Q06187	CHEMBL5251	Kinase	0.106165761464	67/5
Tyrosine-protein kinase Lyn	LYN	P07948	CHEMBL3905	Kinase	0.106165761464	76/6
Fibroblast growth factor receptor 3	FGFR3	P22607	CHEMBL2742	Kinase	0.106165761464	37/3
Platelet-derived growth factor receptor beta	PDGFRB	P09619	CHEMBL1913	Kinase	0.106165761464	114/8
Tyrosine-protein kinase YES	YES1	P07947	CHEMBL2073	Kinase	0.106165761464	34/3
Fibroblast growth factor receptor 2	FGFR2	P21802	CHEMBL4142	Kinase	0.106165761464	35/27
Tyrosine-protein kinase FGR	FGR	P09769	CHEMBL4454	Kinase	0.106165761464	17/4
Macrophage colony stimulating factor receptor	CSF1R	P07333	CHEMBL1844	Kinase	0.106165761464	149/7
Tyrosine-protein kinase BLK	BLK	P51451	CHEMBL2250	Kinase	0.106165761464	20/4
Serine/threonine-protein kinase PLK4	PLK4	O00444	CHEMBL3788	Kinase	0.106165761464	14/2
Ephrin receptor	EPHB4	P54760	CHEMBL5147	Kinase	0.106165761464	30/3
Tyrosine-protein kinase FYN	FYN	P06241	CHEMBL1841	Kinase	0.106165761464	36/3
Dual specificity mitogen-activated protein kinase kinase 2	MAP2K2	P36507	CHEMBL2964	Kinase	0.106165761464	11/3
Tyrosine-protein kinase HCK	HCK	P08631	CHEMBL3234	Kinase	0.106165761464	30/4
Serine/threonine-protein kinase 10	STK10	O94804	CHEMBL3981	Kinase	0.106165761464	13/4
Tyrosine-protein kinase ABL2	ABL2	P42684	CHEMBL4014	Kinase	0.106165761464	13/4
Tyrosine-protein kinase TIE-2	TEK	Q02763	CHEMBL4128	Kinase	0.106165761464	24/16
Ephrin type-A receptor 8	EPHA8	P29322	CHEMBL4134	Kinase	0.106165761464	9/3
Serine/threonine-protein kinase 2	SLK	Q9H2G2	CHEMBL4202	Kinase	0.106165761464	17/4
Tyrosine-protein kinase FRK	FRK	P42685	CHEMBL4223	Kinase	0.106165761464	15/3
Ephrin type-A receptor 6	EPHA6	Q9UF33	CHEMBL4526	Kinase	0.106165761464	10/4
TRAF2- and NCK-interacting kinase	TNIK	Q9UKE5	CHEMBL4527	Kinase	0.106165761464	20/4
Serine/threonine-protein kinase MST1	STK4	Q13043	CHEMBL4598	Kinase	0.106165761464	11/3
Mitogen-activated protein kinase kinase kinase kinase 5	MAP4K5	Q9Y4K4	CHEMBL4852	Kinase	0.106165761464	12/4
Casein kinase I epsilon	CSNK1E	P49674	CHEMBL4937	Kinase	0.106165761464	12/3
Ephrin type-A receptor 3	EPHA3	P29320	CHEMBL4954	Kinase	0.106165761464	11/3
Tyrosine-protein kinase receptor Tie-1	TIE1	P35590	CHEMBL5274	Kinase	0.106165761464	11/4
Mitogen-activated protein kinase kinase kinase kinase 3	MAP4K3	Q8IVH8	CHEMBL5432	Kinase	0.106165761464	14/3
Serine/threonine-protein kinase SIK2	SIK2	Q9H0K1	CHEMBL5699	Kinase	0.106165761464	12/3
Mitogen-activated protein kinase kinase kinase kinase 1	MAP4K1	Q92918	CHEMBL5749	Kinase	0.106165761464	14/3
Serine/threonine-protein kinase 33	STK33	Q9BYT3	CHEMBL6005	Kinase	0.106165761464	16/4
Mitogen-activated protein kinase kinase kinase kinase 4	MAP4K4	O95819	CHEMBL6166	Kinase	0.106165761464	17/4
Serine/threonine-protein kinase TAO2	TAOK2	Q9UL54	CHEMBL1075195	Kinase	0.106165761464	7/3
Serine/threonine-protein kinase TAO3	TAOK3	Q9H2K8	CHEMBL5701	Kinase	0.106165761464	12/2
Voltage-gated calcium channel alpha2/delta subunit 1	CACNA2D1	P54289	CHEMBL1919	Calcium channel auxiliary subunit alpha2delta family	0.106165761464	15/0
Voltage-gated calcium channel alpha2/delta subunit 2	CACNA2D2	Q9NY47	CHEMBL3896	Calcium channel auxiliary subunit alpha2delta family	0.106165761464	4/0
Receptor protein-tyrosine kinase erbB-4	ERBB4	Q15303	CHEMBL3009	Kinase	0.106165761464	29/3
Mitogen-activated protein kinase kinase kinase 8	MAP3K8	P41279	CHEMBL4899	Kinase	0.106165761464	32/0
Serine/threonine-protein kinase/endoribonuclease IRE1	ERN1	O75460	CHEMBL1163101	Enzyme	0.106165761464	18/1
Macrophage-stimulating protein receptor	MST1R	Q04912	CHEMBL2689	Kinase	0.106165761464	8/11
Serine/threonine-protein kinase Aurora-C	AURKC	Q9UQB9	CHEMBL3935	Kinase	0.106165761464	15/2
Fibroblast growth factor receptor 4	FGFR4	P22455	CHEMBL3973	Kinase	0.106165761464	15/2
Ephrin type-A receptor 7	EPHA7	Q15375	CHEMBL4602	Kinase	0.106165761464	7/3
Dual specificity mitogen-activated protein kinase kinase 5	MAP2K5	Q13163	CHEMBL4948	Kinase	0.106165761464	11/4
Serine/threonine-protein kinase RIPK2	RIPK2	O43353	CHEMBL5014	Kinase	0.106165761464	6/4
Discoidin domain-containing receptor 2	DDR2	Q16832	CHEMBL5122	Kinase	0.106165761464	13/3
Activin receptor type-1B	ACVR1B	P36896	CHEMBL5310	Kinase	0.106165761464	6/1
Epithelial discoidin domain-containing receptor 1	DDR1	Q08345	CHEMBL5319	Kinase	0.106165761464	14/4
Mitogen-activated protein kinase kinase kinase kinase 2	MAP4K2	Q12851	CHEMBL5330	Kinase	0.106165761464	15/3
Misshapen-like kinase 1	MINK1	Q8N4C8	CHEMBL5518	Kinase	0.106165761464	14/4
Leukocyte tyrosine kinase receptor	LTK	P29376	CHEMBL5627	Kinase	0.106165761464	10/3
Serine/threonine-protein kinase 35	STK35	Q8TDR2	CHEMBL5651	Kinase	0.106165761464	9/4
Ephrin type-A receptor 1	EPHA1	P21709	CHEMBL5810	Kinase	0.106165761464	9/3
Ephrin receptor	EPHB6	O15197	CHEMBL5836	Unclassified protein	0.106165761464	11/4
Receptor tyrosine-protein kinase erbB-3	ERBB3	P21860	CHEMBL5838	Kinase	0.106165761464	4/3
Serine/threonine-protein kinase MST4	STK26	Q9P289	CHEMBL5941	Kinase	0.106165761464	12/2
Eukaryotic translation initiation factor 2-alpha kinase 1	EIF2AK1	Q9BQI3	CHEMBL6029	Kinase	0.106165761464	3/3
SPS1/STE20-related protein kinase YSK4	MAP3K19	Q56UN5	CHEMBL6191	Kinase	0.106165761464	15/4
Serine/threonine-protein kinase AKT2	AKT2	P31751	CHEMBL2431	Kinase	0.106165761464	318/0
Protein kinase C gamma	PRKCG	P05129	CHEMBL2938	Kinase	0.106165761464	38/0
Serine/threonine-protein kinase AKT	AKT3	Q9Y243	CHEMBL4816	Kinase	0.106165761464	73/0
Serine/threonine-protein kinase PIM1	PIM1	P11309	CHEMBL2147	Kinase	0.106165761464	638/0
Serine/threonine-protein kinase PIM2	PIM2	Q9P1W9	CHEMBL4523	Kinase	0.106165761464	448/0
Serine/threonine-protein kinase PIM3	PIM3	Q86V86	CHEMBL5407	Kinase	0.106165761464	331/0
Cyclin-dependent kinase 2/cyclin E1	CCNE1 CDK2	P24864 P24941	CHEMBL1907605	Kinase	0.106165761464	74/0
Tyrosine-protein kinase JAK1	JAK1	P23458	CHEMBL2835	Kinase	0.106165761464	137/0
Dipeptidyl peptidase VIII	DPP8	Q6V1X1	CHEMBL4657	Protease	0.106165761464	346/0
Dipeptidyl peptidase IX	DPP9	Q86TI2	CHEMBL4793	Protease	0.106165761464	239/0
Phosphodiesterase 4B	PDE4B	Q07343	CHEMBL275	Phosphodiesterase	0.106165761464	43/0
Protein kinase C iota	PRKCI	P41743	CHEMBL2598	Kinase	0.106165761464	287/0
Cyclin-dependent kinase 2	CDK2	P24941	CHEMBL301	Kinase	0.106165761464	170/0
Cyclin-dependent kinase 1	CDK1	P06493	CHEMBL308	Kinase	0.106165761464	146/0
Cyclin T1	CCNT1	O60563	CHEMBL2108	Other cytosolic protein	0.106165761464	111/0
Telomerase reverse transcriptase	TERT	O14746	CHEMBL2916	Enzyme	0.106165761464	79/0
Sodium/calcium exchanger 1	SLC8A1	P32418	CHEMBL4076	Electrochemical transporter	0.106165761464	44/0
Gonadotropin-releasing hormone receptor	GNRHR	P30968	CHEMBL1855	Family A G protein-coupled receptor	0.106165761464	431/0
Amine oxidase, copper containing	AOC3	Q16853	CHEMBL3437	Enzyme	0.106165761464	19/0

**TABLE 3:** Lung fibrosis-related targets obtained from the GeneCards database.

Number	Gene	Number	Gene	Number	Gene	Number	Gene
1	CFTR	335	PDGFA	669	CPLANE1	1,003	HSPH1
2	TGFB1	336	MIR197	670	MKI67	1,004	MT-ATP6
3	TERT	337	MIRLET7B	671	TBX1	1,005	MIR212
4	TP53	338	MIR195	672	PI3	1,006	CASP10
5	TNF	339	MIR96	673	BMP4	1,007	PDE5A
6	SFTPC	340	CHI3L1	674	PMS2	1,008	SH2D1A
7	EGFR	341	MIRLET7E	675	SLC7A7	1,009	SCN5A
8	IL6	342	MIR16-1	676	E2F1	1,010	RSPH4A
9	MUC5B	343	MIR199A1	677	S100A1	1,011	PIK3R2
10	RTEL1	344	MIR210	678	TKT	1,012	DNAH9
11	IL10	345	MIR20A	679	MYRF	1,013	PSMA7
12	SFTPB	346	MIR378A	680	LAMA5	1,014	KIF3A
13	SFTPA1	347	THBD	681	COL2A1	1,015	CYP3A5
14	ELN	348	DMBT1	682	DEFB1	1,016	LOC111674466
15	ABCA3	349	MIR185	683	RPL5	1,017	ADM
16	CAV1	350	IL9	684	SOD1	1,018	PSMC3
17	IFNG	351	CFM1	685	PLK1	1,019	WDPCP
18	MUC1	352	CD79A	686	SCGB3A2	1,020	MIR101-1
19	SFTPA2	353	DYNC2LI1	687	SLC9A3R1	1,021	PRKCB
20	SERPINA1	354	NEK1	688	IREB2	1,022	IL13RA2
21	STAT3	355	IQCB1	689	LOC113664106	1,023	MYH11
22	TERC	356	MIR204	690	FADD	1,024	PRKG2
23	CCN2	357	HLA-A	691	CYP3A4	1,025	SUFU
24	IL13	358	IGF2	692	HSPB1	1,026	PIK3C2A
25	TLR4	359	FLNA	693	MAPK14	1,027	PSMC5
26	PARN	360	BCL2	694	CTSB	1,028	KCNQ1OT1
27	CTNNB1	361	GSN	695	IGF2R	1,029	MIR133A1
28	CCR6	362	MAP2K2	696	BAP1	1,030	GOPC
29	AKT1	363	BPIFA1	697	GATA2	1,031	TRAF2
30	MIR21	364	MIR15A	698	MMP8	1,032	TNFRSF6B
31	NKX2-1	365	EPHX1	699	ITGAV	1,033	MIR139
32	MMP1	366	HPS5	700	PTX3	1,034	LOC111674470
33	SMAD4	367	TRAF3IP1	701	MSLN	1,035	IGHMBP2
34	ACE	368	CFTR-AS1	702	AFF4	1,036	PSMA3
35	SPP1	369	AP3B1	703	CTCF	1,037	PSMC6
36	CXCL8	370	GAPDH	704	ADA	1,038	GADD45B
37	EGF	371	KRT19	705	KITLG	1,039	FKRP
38	FASLG	372	MIR107	706	ZNF423	1,040	DNAAF2
39	CDH1	373	CP	707	IL12A	1,041	ERCC5
40	KRAS	374	HLA-B	708	PRKCA	1,042	E2F3
41	SERPINE1	375	MMP12	709	EGR1	1,043	PIK3R3
42	BRAF	376	AGER	710	DLL4	1,044	PRF1
43	SMAD3	377	DDR1	711	IFNA1	1,045	IFNAR1
44	PRTN3	378	TNNT2	712	EZR	1,046	PF4
45	PIK3CA	379	CCL18	713	LGALS3	1,047	MIR27B
46	IL1RN	380	MIR148B	714	AFP	1,048	VTN
47	HMOX1	381	MIR141	715	TUBB2B	1,049	DNAJC5
48	IL1B	382	INS	716	EPCAM	1,050	BGLAP
49	HLA-DRB1	383	GLI3	717	APOB	1,051	MIR455
50	FAM13A	384	JAK2	718	SPPL2C	1,052	LOC111674471
51	FAS	385	EVC	719	CDK2	1,053	CSF3R
52	PTEN	386	HPS3	720	GDF2	1,054	VCL
53	SFTPD	387	IL33	721	EPO	1,055	NAGLU
54	ITGAM	388	NOS2	722	VCP	1,056	GUSB
55	DSP	389	SLPI	723	HSPA8	1,057	NEAT1
56	STN1	390	MIR133B	724	ATP4A	1,058	TBX4
57	TGFBR1	391	TCTN3	725	HBB	1,059	PSMD1
58	SRC	392	TLR5	726	SELE	1,060	PSMD12
59	CDKN2A	393	MUC4	727	NAT2	1,061	GAST
60	ERBB2	394	MIR93	728	FGF10	1,062	MIR99A
61	CTLA4	395	MIR18A	729	BMP7	1,063	CREB1
62	NFE2L2	396	SLC11A1	730	ETS1	1,064	TRPC6
63	MMP9	397	PSMA6	731	DEFB4A	1,065	EPAS1
64	MTOR	398	RNASE3	732	PRDM10	1,066	MIR125B1
65	HRAS	399	SMAD2	733	GJA1	1,067	IFNGR1
66	TINF2	400	ICAM1	734	RPS6KB1	1,068	PGR
67	FGFR1	401	TGFA	735	JAK3	1,069	TLR1
68	COL1A1	402	RPGR	736	TP63	1,070	CD40
69	NPHP3	403	ANXA5	737	HNF1B	1,071	DLK1
70	F2	404	CXCR1	738	BBS10	1,072	FOS
71	DPP9	405	IL2	739	ITGA2	1,073	DNAL1
72	TGFB2	406	GREM1	740	HOTAIR	1,074	MAD1L1
73	ATP11A	407	CD274	741	SGK1	1,075	PSMD11
74	HFE	408	MIR142	742	DNAAF1	1,076	HBA2
75	PDGFRB	409	GSTP1	743	DNMT1	1,077	RAB11B
76	ITGA3	410	PHOX2A	744	DAB2	1,078	CCKBR
77	MIRLET7D	411	SOS2	745	CDH2	1,079	CLCN3
78	MBL2	412	LMNA	746	HJV	1,080	PPARA
79	NOTCH1	413	ENPP2	747	PRKAA1	1,081	MB
80	MET	414	TOP1	748	HSPA5	1,082	GAS5-AS1
81	TIMP1	415	APEX1	749	CHUK	1,083	LOC111674474
82	SERPINH1	416	CXCL9	750	SKP2	1,084	KCNJ1
83	SCNN1A	417	PLAU	751	ILK	1,085	KIAA0319L
84	MMP2	418	MIRLET7C	752	ADORA2B	1,086	PSMA2
85	ELANE	419	MIR30E	753	SST	1,087	PSMD3
86	VEGFA	420	MIR214	754	CFLAR	1,088	LOC111674473
87	PLG	421	MIR486-1	755	CCNB1	1,089	ABCF2
88	MIR130A	422	KRT7	756	ADCY10	1,090	PSMD8
89	ADRB2	423	TNFRSF13B	757	HDAC9	1,091	MIR590
90	KIT	424	MIR106B	758	ARG1	1,092	PSMD7
91	CC2D2A	425	MIR181A1	759	BAK1	1,093	RNF5
92	TGFBR2	426	MTHFR	760	DICER1	1,094	GLIS3
93	PDGFRA	427	BTNL2	761	IKBKG	1,095	CHAT
94	IL4	428	PTPN11	762	MYH6	1,096	LOC111674476
95	NHP2	429	KDR	763	COL4A3	1,097	MIR23B
96	STK11	430	ELMOD2	764	FGA	1,098	MIR130B
97	NOP10	431	STING1	765	ABL1	1,099	MIR151A
98	ALOX5	432	THY1	766	GJA5	1,100	LOC111674478
99	TMEM67	433	MIR140	767	ARHGAP31	1,101	CD82
100	CCL2	434	VCAM1	768	FCGR3B	1,102	E2F2
101	MIR34C	435	CCR2	769	SLC26A9	1,103	RNF185
102	STAT1	436	CDKN1A	770	LRBA	1,104	MIR423
103	ALB	437	STAT4	771	PMM2	1,105	DEFB103B
104	HGF	438	CEP164	772	ACD	1,106	NFKB2
105	RPGRIP1L	439	MIR128-2	773	CHIT1	1,107	MIR186
106	NPHP1	440	S100A9	774	CDK6	1,108	AFAP1-AS1
107	TSC2	441	TLR3	775	CSF1	1,109	PSMC2
108	MDM2	442	SERPINA3	776	ABCG2	1,110	ERLIN2
109	TTC21B	443	MIR25	777	CASP1	1,111	GADD45G
110	EDN1	444	CD44	778	P2RX7	1,112	CS
111	WDR19	445	MALAT1	779	ASCL1	1,113	CD22
112	CRP	446	COL3A1	780	VWF	1,114	PSMB1
113	CCL11	447	CD28	781	PROM1	1,115	LOC111674479
114	AGT	448	MIR23A	782	PDPN	1,116	LOC111674467
115	CCND1	449	MIR19A	783	PRODH	1,117	SDHB
116	COL1A2	450	HAMP	784	ALG9	1,118	LOC111674465
117	COPA	451	MIR429	785	HOXD13	1,119	PSMD14
118	CASP8	452	MIR335	786	TNFRSF10A	1,120	IFNB1
119	DKC1	453	TTR	787	IL12B	1,121	PLAT
120	ACTC1	454	C2CD3	788	BBS9	1,122	PSMA5
121	FGFR2	455	NR1H4	789	NR3C1	1,123	NEB
122	IL17A	456	G6PD	790	MIR122	1,124	CHGA
123	SCNN1B	457	MIR127	791	ITGB3	1,125	MIR187
124	ATM	458	TNNI3	792	ERF	1,126	LOC113523647
125	MIR155	459	CXCL1	793	CCR5	1,127	HSD3B7
126	SHH	460	MIR424	794	ADIPOQ	1,128	LOC108491823
127	RB1	461	IFT52	795	TF	1,129	IL4R
128	CSF2	462	ZCCHC8	796	RXRA	1,130	PSMB4
129	CEP290	463	AGTR2	797	FAT4	1,131	DRC1
130	FCGR2A	464	NSMCE3	798	PSMD4	1,132	MIR361
131	PKHD1	465	TYR	799	UMOD	1,133	COL11A2
132	NPHP4	466	ABCB4	800	TMEM107	1,134	MIR497
133	MIR200B	467	NOS3	801	BLOC1S6	1,135	SBDS
134	MKS1	468	LPAR1	802	MMP14	1,136	TLR6
135	SCNN1G	469	CXCR2	803	PTK2B	1,137	MIR339
136	FAM111B	470	ZIC3	804	PIEZO2	1,138	RECQL4
137	FN1	471	LOC111674475	805	CDK1	1,139	DERL2
138	INVS	472	CCR7	806	SIRT3	1,140	SH2B3
139	STAT6	473	ANKS6	807	IL15	1,141	GUCA2A
140	WRAP53	474	CALR	808	TAP1	1,142	CCDC103
141	FGFR3	475	TCTN1	809	GATA6	1,143	PCNA
142	RET	476	MIR224	810	EPOR	1,144	PSME2
143	NRAS	477	IGF1R	811	PVT1	1,145	NCOR1
144	DDR2	478	XRCC1	812	MUSK	1,146	TTC37
145	FBN1	479	CD34	813	ESR2	1,147	PSMB5
146	OFD1	480	EVC2	814	MAP1B	1,148	PRKG1
147	MAP2K1	481	CEACAM5	815	MT-CYB	1,149	LEPR
148	ENG	482	MIR338	816	TRAF6	1,150	TNC
149	NEK8	483	GATA4	817	PKD1L1	1,151	PTGS1
150	FGF2	484	MIRLET7G	818	RBPJ	1,152	IFNA2
151	MUC5AC	485	SOD2	819	DMD	1,153	DZIP1L
152	NFKB1	486	MIR191	820	FABP4	1,154	MIR296
153	RARB	487	CCR3	821	CD80	1,155	CD86
154	TNFRSF1A	488	CXCL5	822	PSMA4	1,156	MYCL
155	IL5	489	MYLK	823	IGHE	1,157	VPS45
156	APC	490	H2AC18	824	EGFR-AS1	1,158	GBE1
157	SMPD1	491	GPC3	825	DNMT3B	1,159	IL7R
158	MPO	492	TMEM138	826	MIR26A1	1,160	CYP27A1
159	ACTA2	493	MIR377	827	FLT4	1,161	PLA2G7
160	MIR126	494	CCL22	828	CCNA2	1,162	NOTCH2
161	NOD2	495	SLC34A2	829	DNAI2	1,163	CEACAM1
162	MIR17	496	PTPN22	830	TUG1	1,164	MIR485
163	FGF7	497	PKD2	831	IFIH1	1,165	LOC113633876
164	IFT140	498	DNAH5	832	KATNIP	1,166	REG3A
165	KRT18	499	S100A4	833	MMP3	1,167	EPRS1
166	REN	500	BAX	834	KLF6	1,168	LOC113633875
167	PDCD1	501	THPO	835	BLOC1S3	1,169	LOC113604967
168	VEGFC	502	MIR137	836	SERPINB1	1,170	ZEB1
169	IFT80	503	FOXJ1	837	GLA	1,171	FOXM1
170	SCGB1A1	504	BCL2L1	838	LPO	1,172	CASR
171	BRCA2	505	TRPV4	839	F2R	1,173	TALDO1
172	AHI1	506	CCR4	840	BTK	1,174	MIR219A1
173	TCTN2	507	CADM1	841	SOCS3	1,175	CCL26
174	BMPR2	508	TMEM237	842	COL4A2	1,176	LOC113664107
175	RAF1	509	LOC111674463	843	NBAS	1,177	CLCN4
176	CXCR4	510	GBA	844	CEP104	1,178	SNHG1
177	KIAA0586	511	EZH2	845	MAGEA4	1,179	LAMA2
178	HIF1A	512	SOX9	846	DERL1	1,180	TCF7
179	FARSB	513	MIR24-2	847	AKT3	1,181	TFRC
180	TLR2	514	SPARC	848	NPPA	1,182	PLCZ1
181	AGTR1	515	S100A8	849	BCL2L11	1,183	POMC
182	BMP6	516	LTA	850	LAMA1	1,184	ABCC3
183	CDK4	517	MKKS	851	IL3	1,185	CRYAB
184	TMEM216	518	HYDIN	852	ITGA5	1,186	LOC110806263
185	RELA	519	GLI1	853	CYP2D6	1,187	MCM4
186	CSF3	520	BIRC5	854	DOCK6	1,188	TNFSF13B
187	CCL5	521	MAPK3	855	CANX	1,189	MIR149
188	ESR1	522	PSMB8	856	AXIN2	1,190	PLA2G6
189	MIR223	523	AP3D1	857	SCARB2	1,191	KNG1
190	FOXP3	524	MIRLET7A3	858	CDX2	1,192	HOTTIP
191	TTN	525	IDH1	859	SLC9A3	1,193	AR
192	MARS1	526	CYP2E1	860	ICOS	1,194	PSMB2
193	MAPK8	527	TNFSF10	861	PTGER4	1,195	LSM1
194	MEG3	528	PRKCD	862	SLC17A5	1,196	NIPBL
195	CCL3	529	MIR181C	863	CXCR5	1,197	PSMA8
196	HPS4	530	CYCS	864	TCTEX1D2	1,198	IL16
197	BRCA1	531	IFT27	865	MXRA5	1,199	CCAT1
198	IL2RA	532	TUBB3	866	FOXP1	1,200	SPRY2
199	HPS1	533	HP	867	MIR138-1	1,201	RIPK1
200	MIR34A	534	ALMS1	868	CLDN1	1,202	PHKG2
201	MIR200C	535	NOX4	869	MIR154	1,203	MIR503
202	B9D1	536	RYR1	870	INSR	1,204	GSTT1
203	Dnase1	537	TSLP	871	UCHL1	1,205	PRKCI
204	TSC1	538	VDAC1	872	DCN	1,206	AGL
205	WDR35	539	U2AF1	873	XPNPEP3	1,207	RAG2
206	WT1	540	CAT	874	KRT13	1,208	HNF1A-AS1
207	INPP5E	541	CALCA	875	COMT	1,209	CCAT2
208	MPL	542	DNAH11	876	EDNRA	1,210	ERLIN1
209	MIR125A	543	NF1	877	CA4	1,211	MIR125B2
210	CSPP1	544	PTK2	878	HLA-DQA1	1,212	MIR15B
211	GRP	545	SP110	879	BBS12	1,213	CREBBP
212	MIR144	546	IL12RB1	880	KRT8	1,214	CTSL
213	RTEL1-TNFRSF6B	547	NQO1	881	CMA1	1,215	NR5A1
214	SERPINC1	548	GAA	882	AKR1B10	1,216	MSR1
215	CD4	549	MIR409	883	LOC113633877	1,217	UTP4
216	DYNC2H1	550	VIP	884	CEP55	1,218	MIR301A
217	MIR145	551	GPT	885	MAP2K4	1,219	MUC7
218	FLT1	552	MIR708	886	CDK5	1,220	CASC2
219	TNFRSF1B	553	NFKBIA	887	P2RY2	1,221	ZNRD1ASP
220	MIR29A	554	KEAP1	888	NLRP3	1,222	PLCG2
221	HLA-DQB1	555	LOC111674477	889	PSMC4	1,223	MIR124-1
222	FHIT	556	CD36	890	HSPA4	1,224	MIR382
223	H19	557	IGFBP3	891	AVPR2	1,225	LAMP1
224	PKD1	558	NKX2-5	892	RPS27A	1,226	CD69
225	SOS1	559	CEP83	893	MIR375	1,227	DANCR
226	SPINK1	560	BDNF	894	MIR33A	1,228	TP53COR1
227	MIR31	561	ABCB11	895	ATF6	1,229	MYL3
228	IFT172	562	FGFR4	896	RXRB	1,230	CYSLTR2
229	MMP7	563	CTSG	897	UBC	1,231	SELL
230	CD8A	564	ITGB1	898	NPC2	1,232	LINC-ROR
231	PDGFB	565	CSF2RA	899	CLCN2	1,233	NOS1
232	SLC2A1	566	DNAI1	900	FOXE1	1,234	IFT20
233	JUN	567	ARL3	901	MIR132	1,235	MIR10B
234	BMP2	568	MECP2	902	HDGF	1,236	CD81
235	HMGB1	569	CDKN1B	903	WNT7B	1,237	ANTXR1
236	ERBB3	570	DNMT3A	904	SLC9A3R2	1,238	PRSS2
237	MIR150	571	VHL	905	H2AX	1,239	SNHG20
238	PRSS1	572	THBS1	906	PRKAG2	1,240	MIR95
239	MIR29C	573	YAP1	907	SERPINB3	1,241	GALC
240	MIR146B	574	TIMP2	908	LRP1B	1,242	DGCR5
241	ABCC1	575	TOLLIP	909	DNAAF3	1,243	HNMT
242	BBS2	576	KCNK3	910	BCL10	1,244	SLCO2A1
243	ABCB1	577	NOTCH3	911	RAG1	1,245	MLH1
244	CXCL10	578	RASSF1	912	LAMA4	1,246	PLA2G2A
245	XIAP	579	SP1	913	HLA-G	1,247	MME
246	KIF21A	580	RHOA	914	PSMB3	1,248	TYMS
247	PIK3R1	581	ABCC2	915	SIRT1	1,249	MIR198
248	BBS1	582	IKBKB	916	MIRLET7A1	1,250	JAK1
249	IRF1	583	GZMB	917	PLA2G1B	1,251	PDE4D
250	IL1A	584	LEP	918	KDM4C	1,252	LAMC2
251	PTPRC	585	BBS5	919	MUC6	1,253	AHR
252	HLA-DPB1	586	APOE	920	TUBB1	1,254	TPM1
253	ERCC6	587	NHLRC2	921	SKIV2L	1,255	MT-CO2
254	STX1A	588	NPPB	922	TPM2	1,256	ASCC1
255	SETD2	589	SYP	923	CCDC40	1,257	EOGT
256	CCL17	590	ACTB	924	TET2	1,258	CCL7
257	IDH2	591	PSMA1	925	TRIP11	1,259	TRPM4
258	CXCL12	592	PTCH1	926	ITGA2B	1,260	GPSM2
259	MIR27A	593	LOXL2	927	ENO2	1,261	TARS1
260	GSTM1	594	CEP41	928	EIF2AK3	1,262	TPM3
261	DCTN4	595	MYPN	929	RSPH9	1,263	CD14
262	PRKN	596	CR1	930	MIR193A	1,264	MIR216A
263	CXCR3	597	ERCC1	931	PXN	1,265	CTAG1B
264	MIR200A	598	IGFBP5	932	DNAAF5	1,266	MIR22HG
265	ARL13B	599	MGMT	933	LAT	1,267	MIR10A
266	PTGS2	600	VIM	934	RSPH1	1,268	PCAT1
267	MIR148A	601	ENO1	935	CDKN2B-AS1	1,269	SNHG15
268	MYC	602	NLRC4	936	ADORA1	1,270	BANCR
269	IGF1	603	PIK3CG	937	RAD51	1,271	PSAP
270	IL18	604	MYH7	938	DIABLO	1,272	IL11
271	CYP1A1	605	F13A1	939	TFR2	1,273	MIR362
272	NEK9	606	WNT4	940	RSPO2	1,274	UCA1
273	LOX	607	NME1	941	C4A	1,275	UBE2L3
274	MIR30D	608	CD63	942	IFI27	1,276	FBLN5
275	MIR146A	609	RPGRIP1	943	MIR29B1	1,277	GC
276	IFT122	610	ATP8B1	944	HSPD1	1,278	TP73-AS1
277	B9D2	611	GNAS	945	CCDC39	1,279	PPBP
278	KCNQ1	612	TUBB	946	PDE4A	1,280	LINC00473
279	CYP2A6	613	IFRD1	947	TRIM21	1,281	SOX2-OT
280	MIR483	614	CASP9	948	PLCG1	1,282	MIR181B1
281	MIR183	615	LAMP2	949	IL1RL1	1,283	XIST
282	IL1R1	616	HDAC2	950	BBIP1	1,284	MIR129-1
283	SDCCAG8	617	ANXA1	951	MIR196A1	1,285	PRL
284	PTRH2	618	IFT74	952	BAD	1,286	MIR193B
285	MIR182	619	ROS1	953	MYBPC3	1,287	NR3C2
286	CDKN3	620	CLEC7A	954	ARAF	1,288	MYL1
287	GUCY2C	621	AKT2	955	VDR	1,289	SDC1
288	MIR222	622	WRN	956	MAGEA1	1,290	MIR24-1
289	LOC111674472	623	DTNBP1	957	TAC1	1,291	PHB
290	KIF7	624	CD19	958	IDUA	1,292	MYL2
291	FLNC	625	SMARCA4	959	EPX	1,293	APOA1
292	PPARG	626	ANGPT2	960	ASXL1	1,294	SNHG12
293	XRCC3	627	PARP1	961	MAGEA3	1,295	CEP57
294	SNAI1	628	BBS7	962	RAC1	1,296	IL6R
295	POSTN	629	MIF	963	GATA1	1,297	SERPINF2
296	MIR192	630	SELP	964	GAS5	1,298	CALB2
297	JAG1	631	HSPG2	965	MIR9-1	1,299	MIR152
298	CD40LG	632	ATP12A	966	RYR2	1,300	ADK
299	DYNC2I1	633	HSP90AA1	967	JPH2	1,301	NRG1
300	ACVRL1	634	CHRM3	968	RIOX2	1,302	MIR501
301	MIR221	635	MVP	969	LOC111674464	1,303	GPRC5A
302	IFT43	636	DCDC2	970	TNFAIP3	1,304	LZTR1
303	DES	637	ERCC2	971	PIK3CB	1,305	TLR7
304	MIR22	638	PPP2R1B	972	DHCR7	1,306	POT1
305	TNFRSF10B	639	TYMP	973	C1S	1,307	EP300
306	IRF5	640	CLCA1	974	SNAI2	1,308	FBL
307	ALK	641	OGG1	975	ERBB4	1,309	PLOD2
308	CEP120	642	MCL1	976	ACVR1	1,310	DLL1
309	MIR451A	643	CCN4	977	NPC1	1,311	TOP2A
310	DYNC2I2	644	F3	978	LNX1	1,312	MAGEC2
311	CASP3	645	STAT5B	979	GRB2	1,313	MIR499A
312	CCL4	646	FUZ	980	TFAP2B	1,314	ENSG00000266919
313	HPS6	647	LBR	981	SLC6A4	1,315	TNFRSF11B
314	MIR143	648	CXCL2	982	IFT88	1,316	HOXA11-AS
315	ICOSLG	649	PLAUR	983	NPHS1	1,317	RASGRP1
316	CLCA4	650	ASAH1	984	MAP2K7	1,318	SPRY4-IT1
317	HLA-DPA1	651	BPI	985	PSMD2	1,319	FIP1L1
318	FOXF1	652	NPM1	986	DNAH8	1,320	GSR
319	BIRC3	653	ACTA1	987	B2M	1,321	CST3
320	SMAD7	654	ANGPT1	988	INTU	1,322	LTBP4
321	MIR203A	655	GGT1	989	ENTPD1	1,323	ZFAS1
322	MIR30A	656	CAMP	990	PRSS8	1,324	F5
323	MIR324	657	SOD3	991	CCNE1	1,325	STMN1
324	MIR199B	658	F2RL3	992	LRRC56	1,326	AIRE
325	ACP5	659	TEK	993	MAP3K8	1,327	RETN
326	MIR205	660	LTF	994	SHC1	1,328	NTS
327	TP73	661	MT-CO1	995	CCDC114	1,329	KRT5
328	TLR9	662	LIPA	996	ACHE	1,330	F2RL1
329	MAPK1	663	WNT3	997	TBX20	1,331	TNFSF11
330	CLCN5	664	GDF1	998	AURKB	1,332	COL4A5
331	TMEM231	665	SLC40A1	999	ASL	1,333	PIK3CD
332	BBS4	666	GLIS2	1,000	MIR30C1	1,334	FGF9
333	RMRP	667	AREG	1,001	IL2RB	1,335	KRT20
334	TGFB3	668	CDKN2B	1,002	NCF2		

**TABLE 4:** Common targets shared between the potential anlotinib targets and lung fibrosis-related targets.

Number	Gene	Number	Gene
1	AXL	38	EPHA3
2	MERTK	39	TIE1
3	ERBB2	40	CACNA2D1
4	AURKB	41	ERBB4
5	FLT1	42	MAP3K8
6	EGFR	43	ERN1
7	KDR	44	MST1R
8	SRC	45	FGFR4
9	MET	46	EPHA7
10	RET	47	MAP2K5
11	ALK	48	RIPK2
12	ABL1	49	DDR2
13	KIT	50	ACVR1B
14	ACVR1	51	DDR1
15	FLT4	52	MAP4K2
16	FLT3	53	EPHA1
17	PDGFRA	54	EPHB6
18	FGFR1	55	ERBB3
19	TGFBR1	56	EIF2AK1
20	BTK	57	MAP3K19
21	LYN	58	AKT2
22	FGFR3	59	PRKCG
23	PDGFRB	60	AKT3
24	YES1	61	PIM1
25	FGFR2	62	PIM2
26	FGR	63	JAK1
27	CSF1R	64	DPP8
28	BLK	65	DPP9
29	PLK4	66	PDE4B
30	FYN	67	PRKCI
31	MAP2K2	68	CDK2
32	HCK	69	CDK1
33	STK10	70	CCNT1
34	ABL2	71	TERT
35	TEK	72	SLC8A1
36	SLK	73	GNRHR
37	STK4	74	AOC3

### PCBP3 Posttranscriptionally Increases PFKFB3 Expression by Promoting Its Translation During Myofibroblast Activation

**FIGURE 3:**
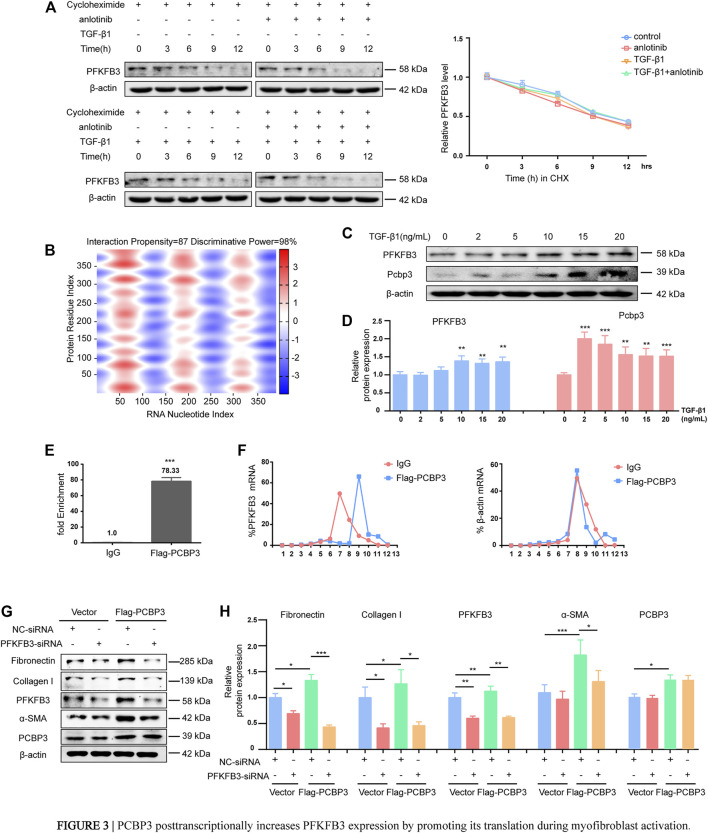
PCBP3 posttranscriptionally increases PFKFB3 expression by promoting its translation during myofibroblast activation. **(A)** PFKFB3 degradation in mouse lung fibroblasts when protein synthesis was inhibited by 50 μM cycloheximide (mean ± SD, n = 3). **(B)** Prediction of RNA–protein interaction of PFKFB3 mRNA with PCBP3 protein using the catRAPID algorithm. Red represents interaction strength. **(C)** Western blot analysis of PFKFB3 and PCBP3 protein levels in mouse lung fibroblasts stimulated with TGF-β1 for the indicated concentrations. **(D)** Quantification of PFKFB3 and PCBP3 protein levels relative to β-actin is shown (mean ± SD, n = 3, **p < 0.01, ***p < 0.001 compared with 0 by one-way ANOVA). **(E)** Quantitative RT-PCR (qPCR) to show the effect of PCBP3 overexpressing on PFKFB3 RNA immunoprecipitation (RIP) in mouse lung fibroblasts. Values were plotted as mean ± SD from three independent experiments. *p* value was calculated by Student t test. ***p < 0.001. **(F)** Mouse lung fibroblasts expressing Flag-PCBP3 were fractionated into cytoplasmic extracts through sucrose gradients. The arrow indicates the direction of sedimentation. The distribution of PFKFB3 and β-actin mRNAs was quantified by RT-PCR analysis of RNA isolated from 12 gradient fractions. Statistical analyses were performed using Student t test. ***p < 0.001. **(G)** Mouse lung fibroblasts were transfected with Flag-PCBP3, and then transfected with PFKFB3-siRNAs or NC-siRNA. The levels of Fibronectin、Collagen I、PFKFB3、α-SMA、PCBP3 and β-actin assessed by western blot. **(H)** Graphical representation of the relative levels of indicated proteins (mean ± SD, n = 3, *p < 0.05, **p < 0.01, ***p < 0.001 by ANOVA).

Interestingly, the progressive upregulation of PFKFB3 during myofibroblast activation induced by TGF-β1 that was observed at the protein level was not confirmed at the mRNA level, as measured by RT-PCR (Supplementary Figures S3A, B). These results indicate that TGF-β1-induced overexpression does not require *de novo* transcription of PFKFB3. To further verify these findings, primary MLFs were incubated with cycloheximide to block new protein synthesis, and immunoblotting was used to measure PFKFB3 levels (Figure 3A). The half-life of PFKFB3 was not significantly altered, indicating that TGF-β1 does not influence PFKFB3 protein stability. Therefore, we postulated that PFKFB3 upregulation is modulated through posttranscriptional mechanisms in this context. To verify this hypothesis, we used the online tool catRAPID to screen for potential proteins that may interact with PFKFB3 mRNA and identified that PCBP3 (Table 5) (Agostini et al., 2013; Livi et al., 2016), a member of the PCBP family, has a high probability of directly interacting with PFKFB3 mRNA (Figure 3B) (Choi et al., 2007; Kang et al., 2012; Leidgens et al., 2013; Wang J. et al., 2020). We comparatively analyzed the expression of PCBP3 after treatment with different doses of TGF-β1 by immunoblot analysis and found that PCBP3 protein expression was increased in primary MLFs after TGF-β1 treatment (Figures 3C,D), which correlated with PFKFB3 overexpression. To better define the connection between PCBP3 function and PFKFB3, we performed RNA-protein coimmunoprecipitation (RIP) studies in primary MLFs transfected with FLAG-tagged PCBP3 (FLAG-PCBP3). An antibody targeting the FLAG protein was used to immunoprecipitate FLAG-PCBP3 and any interacting molecules from the cell lysates. Reverse transcription followed by PCR was then used to identify individual PFKFB3 mRNAs isolated with FLAG-PCBP3. We found that PFKFB3 transcripts were enriched by PCBP3 coimmunoprecipitation compared to control IgG coimmunoprecipitation (Figure 3E), demonstrating that PFKFB3 mRNA is indeed a direct target of PCBP3 in MLFs. To test the possibility that PCBP3 may influence PFKFB3 translation, we performed polysome analysis in cells transfected with FLAG-PCBP3. Cytoplasmic lysates were fractionated through sucrose gradients to separate ribosomal subunits (40S and 60S), monosomes (80S) and progressively larger polysomes. RNA was extracted from each of the 12 fractions, and the levels of PFKFB3 and β-actin mRNA were quantified by quantitative RT-PCR. While PFKFB3 mRNA levels peaked in fraction 7 in control cells, the distribution of PFKFB3 mRNA shifted rightward when PCBP3 was overexpressed, peaking in fraction 9, indicating that PFKFB3 mRNA formed, on average, larger polysomes after PCBP3 overexpression (Figure 3F). The distribution of β-actin mRNA was not affected by PCBP3 overexpression. These results indicated that overexpression of PCBP3 increases the translation of PFKFB3. Overall, these results suggest that PCBP3 improves PFKFB3 expression levels by increasing its translation rather than by influencing its protein stability.

**TABLE 5:** Potential proteins may interact with PFKFB3 mRNA through the catRAPID algorithm.

#	Protein D	RNA ID	Z score^?^	Discriminative power (%)^?^	Interaction strength (%)^?^	Domain^?^	Motif^?^	Ranking^?^
1	ELAV1_MOUSE_247-308	NC_000068.7:c11_1_5924-6036	−0.13	50	98	yes	yes	HHH
2	ELAV1_MOUSE_247-308	NC_000068.7:c11_1_26816-27008	−0.08	67	99	yes	yes	HHH
3	ELAV1_MOUSE_247-308	NC_000068.7:c11_1_15806-15983	−0.23	40	90	yes	yes	HHH
4	PCBP3_MOUSE_301-351	NC_000068.7:c11_1_41382-41564	−0.69	14	13	yes	yes	HHH
5	ELAV1_MOUSE_247-308	NC_000068.7:c11_1_77223-77340	−0.47	22	74	yes	yes	HHH
6	ELAV1_MOUSE_247-308	NC_000068.7:c11_1_10399-10532	−0.50	20	64	yes	yes	HHH
7	ELAV1_MOUSE_109-176	NC_000068.7:c11_1_5924-6036	−0.14	50	98	yes	yes	HHH
8	ELAV1_MOUSE_109-176	NC_000068.7:c11_1_45706-45815	−0.39	26	85	yes	yes	HHH
9	ELAV1_MOUSE_109-176	NC_000068.7:c11_1_26816-27008	−0.04	63	99	yes	yes	HHH
10	ELAV1_MOUSE_109-176	NC_000068.7:c11_1_15806-15983	−0.24	40	90	yes	yes	HHH
11	ELAV1_MOUSE_109-172	NC_000068.7:c11_1_5924-6036	−0.15	47	97	yes	yes	HHH
12	ELAV1_MOUSE_109-172	NC_000068.7:c11_1_45706-45815	−0.40	26	85	yes	yes	HHH
13	ELAV1_MOUSE_109-172	NC_000068.7:c11_1_15806-15983	−0.26	37	87	yes	yes	HHH
14	PCBP3_MOUSE_301-351	NC_000068.7:c11_1_77461-77636	−0.71	14	9	yes	yes	HHH
15	PCBP3_MOUSE_301-351	NC_000068.7:c11_1_66050-66214	−0.66	14	17	yes	yes	HHH

To determine the functional impact of PCBP3-mediated regulation of PFKFB3 expression in lung fibrosis, we transfected lung fibroblasts with FLAG-PCBP3. Expression of PFKFB3 was significantly increased by PCBP3 overexpression compared to that of the empty vector control. Reliable markers of the phenotypic transformation of fibroblasts into myofibroblasts, fibronectin, collagen I and α-SMA, were markedly increased in FLAG-PCBP3-treated cells at the protein level (Figures 3G,H) compared with vector-treated cells. In turn, using small interfering RNA (siRNA) to silence PFKFB3, the FLAG-PCBP3-induced overexpression of fibronectin, collagen I and α-SMA was abolished (Figures 3G,H). These findings suggest that PCBP3 protein upregulation is an early and sustained event during fibroblast activation and that the profibrogenic effects of PCBP3 are mediated by PFKFB3 expression. Taken together, these data suggest that PCBP3 posttranscriptionally increases PFKFB3 expression by promoting its translation during myofibroblast activation.

### Anlotinib Represses PCBP3 Expression Levels During Myofibroblast Activation

**FIGURE 4:**
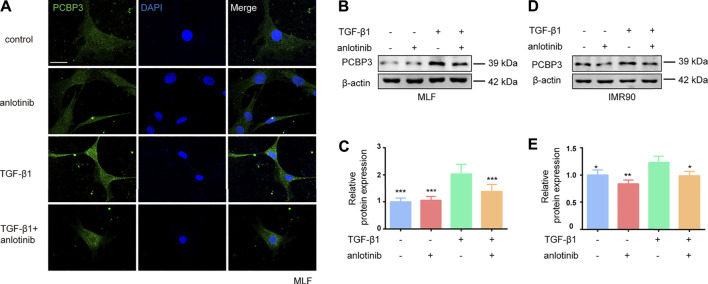
Anlotinib represses PCBP3 expression levels during myofibroblast activation. **(A)** Immunofluorescence for PCBP3 (green) in mouse lung fibroblasts treated with anlotinib for 3  h, followed by TGF-β1 for an additional 24 h. DAPI-stained nuclei (blue). Scale bar, 25 µm. **(B)** Western blots analysis of PCBP3 and β-actin in primary mouse lung fibroblasts. **(C)** Quantification for the indicated proteins (mean ± SD, n = 3). **(D)** The Western blots analysis of PCBP3 and β-actin in IMR90 cells treated with anlotinib for 3  h, followed by TGF-β1 for an additional 24 h. **(E)** Quantification for the indicated proteins (mean ± SD, n = 3). *p < 0.05, **p < 0.01, ***p < 0.001 VS TGF-β1-treated group by ANOVA.

To confirm the regulation of PCBP3 by anlotinib *in vitro*, we evaluated the protein expression of PCBP3 in MLFs and IMR90 cells. We found that TGF-β1 induced the expression of PCBP3 in MLFs and that anlotinib prevented PCBP3 expression by immunofluorescence analysis (Figure 4A). Western blot analysis of PCBP3 showed a similar result (Figures 4B,C) in MLFs, and these results were confirmed in the human IMR90 cell line (Figures 4D,E). Taken together, these data suggest that anlotinib can repress PCBP3 expression levels during myofibroblast activation *in vitro*.

### Anlotinib Attenuates Bleomycin-Induced Pulmonary Fibrosis

**FIGURE 5:**
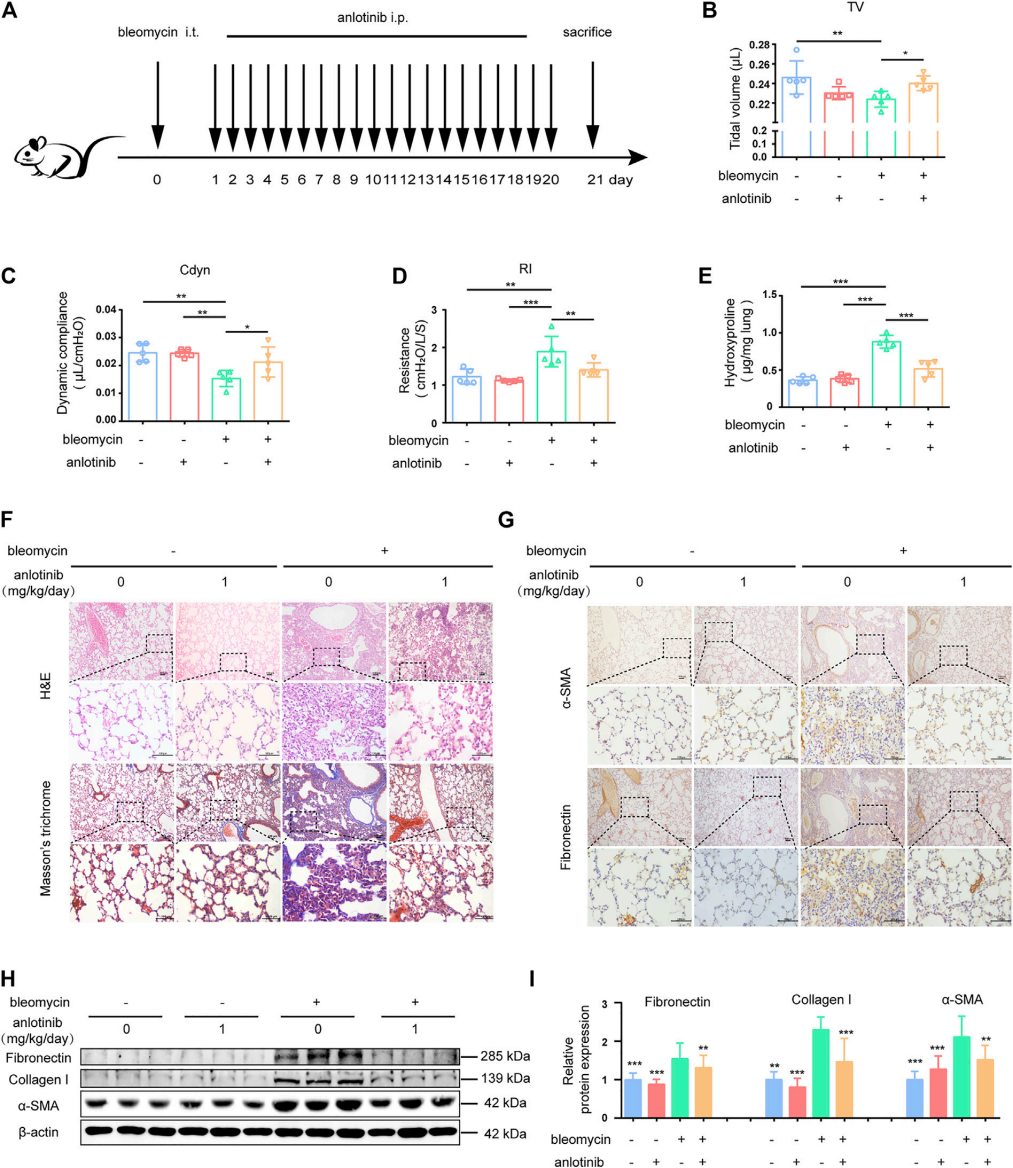
Anlotinib attenuates bleomycin-induced pulmonary fibrosis. **(A)** Intervention dosing regimen of anlotinib in experimental mouse model of fibrosis. C57BL/6 mice were intraperitonealy injuected with 1 mg/kg of anlotinib or vehicle (n = 5-6 per group) daily after bleomycin instillation. Lungs were harvested at 21 days for the following analyses. Analysis of tidal volume (TV) **(B)**, dynamic compliance (Cdyn) **(C)**, and lung resistance (RI) **(D)** (mean ± SD, n = 5). **(E)** Hydroxyproline (HYP) contents in lung tissues from mice (mean ± SD, n = 5). Representative images show haematoxylin and eosin (H&E), Masson’s trichrome **(F)**, α-SMA and Fibronectin staining **(G)** of lung sections from the indicated groups of mice. Scale bars, 100 µm. **(H)** Western blot analysis of Fibronectin、Collagen I and α-SMA, β-actin was used as a loading control. **(I)** Quantification of Fibronectin、Collagen I and α-SMA protein levels relative to β-actin is shown (mean ± SD, n = 6). *p < 0.05, **p < 0.01, ***p < 0.001 VS BLM-treated group by one-way ANOVA.

To investigate the biological effects of anlotinib on pulmonary fibrosis *in vivo*, we established a bleomycin (BLM)-induced mouse model of pulmonary fibrosis. The mice were intraperitoneally injected with 1 mg/kg anlotinib daily after BLM administration (Figure 5A). From the first week after bleomycin instillation, the bleomycin-treated mice showed a certain reduction in activity, accompanied by slight shortness of breath. 21 days after bleomycin administration, bleomycin-treated mice showed obvious hyperventilation, accompanied by reduced activity and weight loss, but no similar symptoms were observed in the control group. A single dose of BLM (5 mg/kg) administered by intratracheal instillation successfully induced pulmonary fibrosis in C57BL/6 mice, as evidenced by a decline in pulmonary function, decreased tidal volume (TV, Figure 5B) and dynamic compliance (Cdyn, Figure 5C), and increased lung resistance (RI, Figure 5D). However, treatment with anlotinib significantly reversed bleomycin-induced pulmonary dysfunction. Moreover, we evaluated collagen deposition in the lung tissues by analyzing the hydroxyproline (HYP) content and found that anlotinib treatment reduced the amount of collagen in the lungs of bleomycin-treated mice (Figure 5E). Hematoxylin and eosin (H&E) staining indicated that anlotinib-treated mice had decreased lung inflammation and reduced lung architectural damage (Figure 5F). Accordingly, Masson’s trichrome staining showed decreased collagen deposition in anlotinib-treated mice compared with vehicle-treated mice (Figure 5F). Furthermore, attenuated fibrosis was supported by decreased protein levels of fibronectin and α-SMA by immunohistochemical (IHC) staining (Figure 5G). We also found that anlotinib treatment reduced fibronectin, collagen I and α-SMA expression by western blotting (Figures 5H,I). Taken together, these data show that anlotinib attenuates bleomycin-induced pulmonary fibrosis *in vivo*.

### Anlotinib Decreases PCBP3 Expression and Inhibits PFKFB3-Driven Glycolysis in Fibrotic Rodent Lungs

**FIGURE 6:**
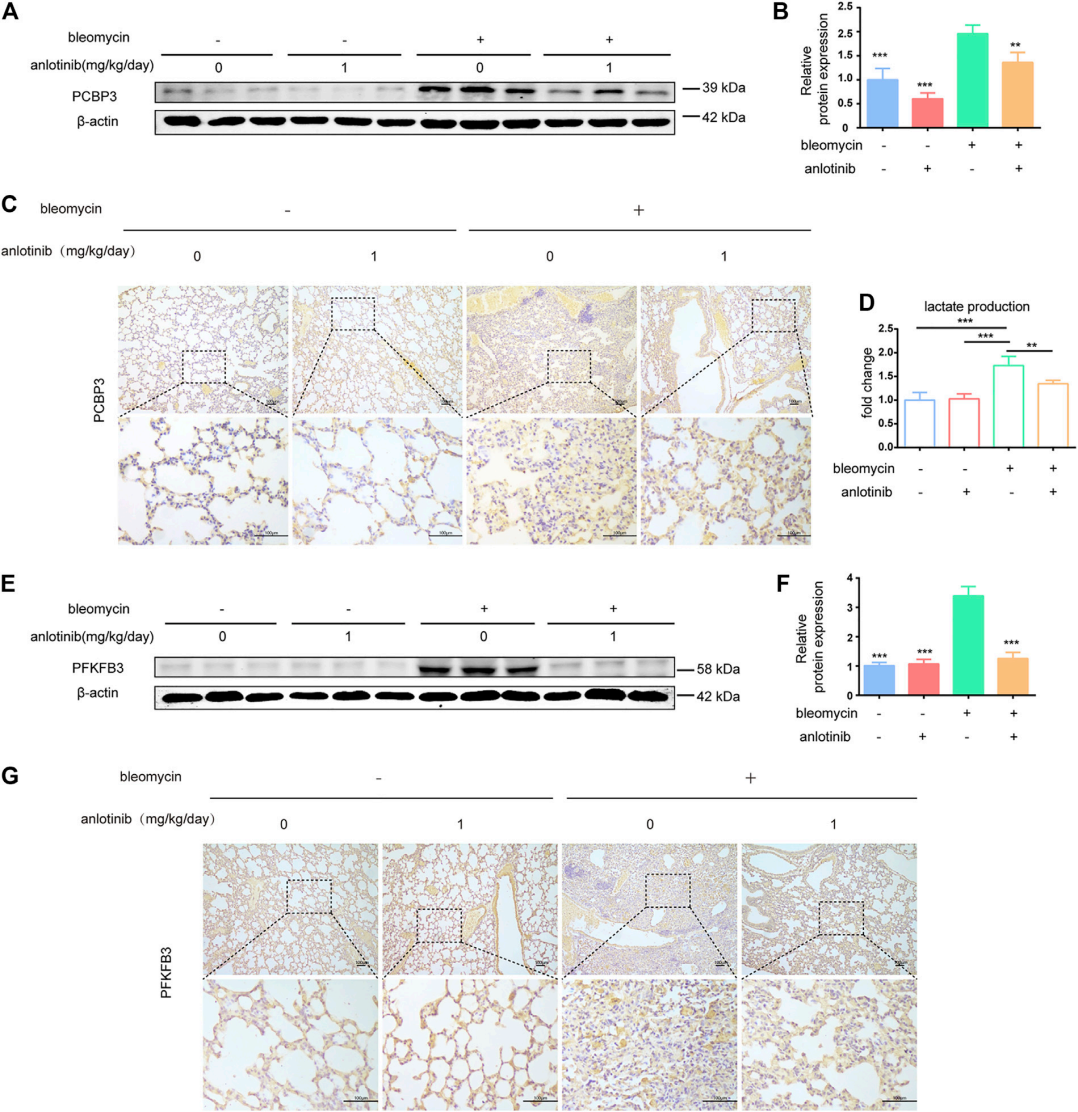
Anlotinib decreases PCBP3 expression and inhibit the PFKFB3-driven glycolysis in fibrotic rodent lungs. **(A)** Intervention dosing regimen of anlotinib in experimental mouse model of fibrosis. C57BL/6 mice were intraperitonealy injuected with 1 mg/kg of anlotinib or vehicle (n = 5-6 per group) daily after bleomycin instillation. Lungs were harvested at 21 days. Western blot analysis of PCBP3, β-actin was used as a loading control (n = 6). **(B)** Quantification of PCBP3 protein levels relative to β-actin is shown (mean ± SD, n = 6). **(C)** Representative images show PCBP3 staining of lung sections from the indicated groups of mice. Scale bars, 100 µm. **(D)** lactate contents in lung tissues from mice (mean ± SD, n = 5). **(E)** Western blot analysis of PFKFB3, β-actin was used as a loading control (n = 6). **(F)** Quantification of PFKFB3 protein levels relative to β-actin is shown (mean ± SD, n = 6). **(G)** Representative images show PFKFB3 staining of lung sections from the indicated groups of mice. Scale bars, 100 µm. **p < 0.01, ***p < 0.001 VS BLM-treated group by one-way ANOVA.

We next examined whether the levels of PCBP3 were regulated by anlotinib *in vivo*. We evaluated the expression of PCBP3 in lung tissues and found that the protein levels of PCBP3 were markedly increased after bleomycin instillation, while anlotinib treatment decreased PCBP3 expression (Figures 6A,B). Accordingly, IHC staining showed decreased PCBP3 protein levels in anlotinib-treated mice compared with vehicle-treated mice (Figure 6C). In addition, to confirm the regulation of PFKFB3-driven glycolysis by anlotinib *in vivo*, we measured the levels of lactate and the expression of PFKFB3 in the lungs of mice. We found that there were significantly higher levels of lactate in the lungs of bleomycin-treated mice than in the lungs of control mice, and anlotinib decreased lactate levels (Figure 6D). Western blot and IHC staining studies revealed that bleomycin-induced PFKFB3 expression in the lungs of mice was prevented by anlotinib (Figures 6E–G). Overall, these results suggest that anlotinib decreases PCBP3 expression and inhibits PFKFB3-driven glycolysis in fibrotic rodent lungs.

### Anlotinib Accelerates the Resolution of Bleomycin-Induced Lung Fibrosis

**FIGURE 7:**
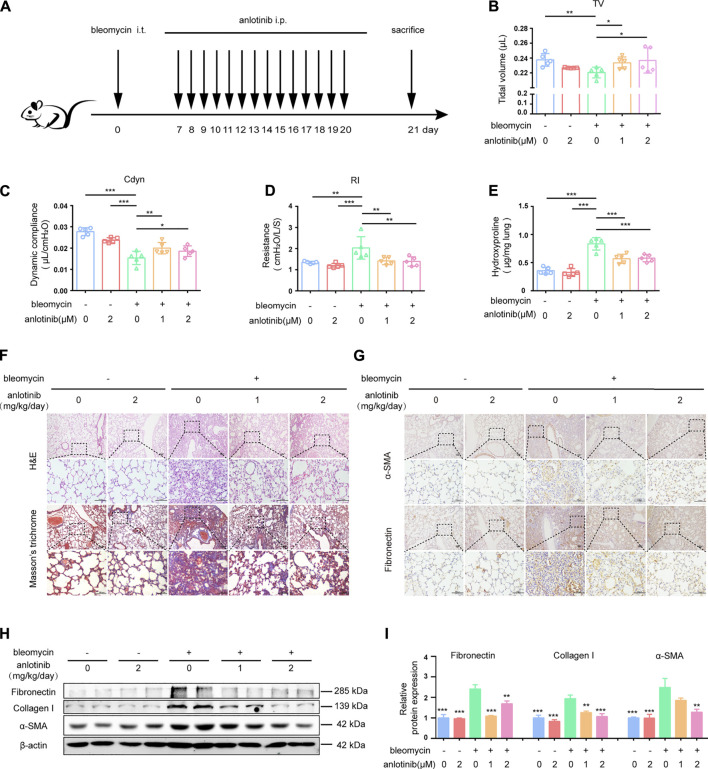
Anlotinib accelerates resolution of bleomycin-induced pulmonary fibrosis. **(A)** Intervention dosing regimen of anlotinib in established pulmonary fibrosis. Bleomycin instillation was used to induce fibrosis and no treatment was given during the first 7 d. Then, mice were intraperitonealy injuected with 1 or 2 mg/kg of anlotinib or vehicle (n = 5-6 per group) daily. Lungs were harvested at 21 days. Tidal volume (TV) **(B)**, dynamic compliance (Cdyn) **(C)**, and lung resistance (RI) **(D)** of mice were measured (mean ± SD, n = 5). **(E)** HYP contents in lung tissues from mice (mean ± SD, n = 5). Representative images show H&E, Masson’s trichrome **(F)**, α-SMA and Fibronectin staining **(G)** of lung sections from the indicated groups of mice. Scale bars, 100 µm. **(H)** Western blot analysis of Fibronectin、Collagen I and α-SMA, β-actin was used as a loading control (n = 4). **(I)** Quantification of Fibronectin、Collagen I and α-SMA protein levels relative to β-actin is shown (mean ± SD, n = 5). *p < 0.05, **p < 0.01, ***p < 0.001 VS BLM-treated group by one-way ANOVA.

We demonstrated that anlotinib treatment could attenuate bleomycin-induced pulmonary fibrosis. In that *in vivo* experiment, anlotinib was administered at approximately the same time as bleomycin instillation. We further examined whether anlotinib could postpone the progression of established fibrosis. Therefore, we performed another *in vivo* experiment in which anlotinib was intraperitoneally injected 7 days after bleomycin instillation (Figure 7A). As interventions beginning 7 days post bleomycin were classified as therapeutic (Izbicki et al., 2002; Moeller et al., 2008), we initially treated mice with anlotinib (1 mg/kg/day or 2 mg/kg/day) beginning on day 7 after bleomycin instillation. Pulmonary function tests showed that anlotinib treatment reversed the bleomycin-induced decline in pulmonary function, with increases in TV (Figure 7B) and Cdyn (Figure 7C) and a decrease in RI (Figure 7D). HYP measurements showed that the collagen content was significantly decreased in anlotinib-treated mice compared with vehicle-treated mice (Figure 7E). H&E staining and Masson’s trichrome staining of lungs collected at day 21 showed enhanced recovery from fibrosis upon anlotinib treatment (Figure 7F). Correspondingly, IHC staining showed that anlotinib treatment reduced fibronectin and α-SMA expression in the lungs (Figure 7G). Western blot analysis also showed that anlotinib decreased the protein levels of fibronectin, collagen I and α-SMA in the lungs (Figures 7H,I). Collectively, these data clearly demonstrate that anlotinib accelerates fibrosis resolution *in vivo* even after the establishment of fibrosis.

## Discussion

**FIGURE 8:**
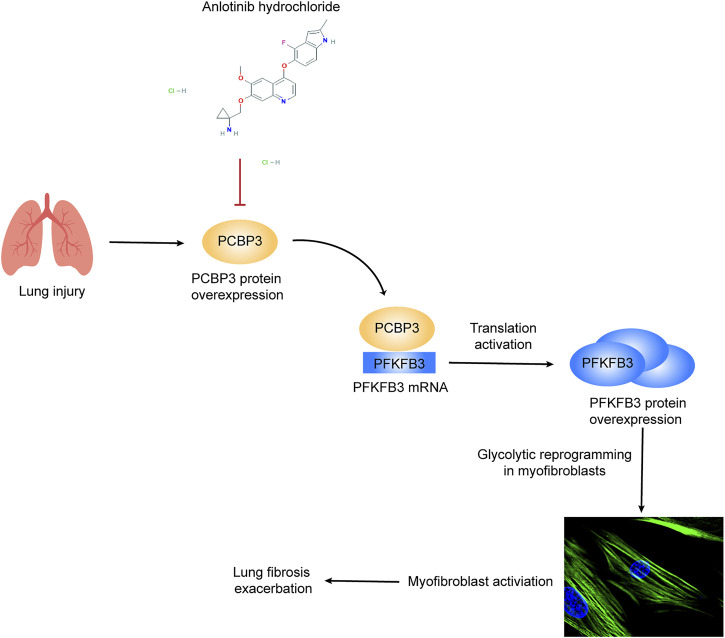
Schematic representation of PCBP3-PFKFB3-dependent glycolysis and the inhibitory effect of anlotinib on this pathway. Lung injury induces PCBP3 expression, which results in an increase in PFKFB3 expression by promoting its translation, resulting in the augmentation of glycolysis in lung fibroblasts. Glycolytic reprogramming participates in myofibroblast activation and furthers lung fibrosis. The tyrosine kinase inhibitor anlotinib inhibits PFKFB3-driven glycolysis by decreasing the expression of PCBP3, thereby suppressing myofibroblast activation and inhibiting the exacerbation of lung fibrosis.

Despite recent advances in our understanding of IPF pathology, there is still no curative treatment for this disease; indeed, the currently available antifibrotic treatment modalities slow but do not completely stop the progression of the disease (Spagnolo and Maher, 2017). In this study, we demonstrate that anlotinib strongly inhibits fibroblast-to-myofibroblast transdifferentiation and reduces extracellular matrix production in primary MLFs and in the human IMR90 cell line. Accordingly, preventative and therapeutic administration of anlotinib to bleomycin-administered mice resulted in accelerated resolution of fibrosis. No adverse, systemic side effects were observed. Here, we demonstrate a novel mechanism by which anlotinib exerts antifibrotic effects by downregulating PCBP3, reducing PFKFB3 translation and inhibiting glycolysis in myofibroblasts (Figure 8).

A previous study revealed that male and female C57BL/6 mice did not differ in terms of their lung fibrotic responses, including cellular infiltration, collagen deposition, and quantifiable morphological changes in the lung architecture, but that the bleomycin-induced decrease in static compliance was significantly greater in males than in females (Voltz et al., 2008). This adverse effect on lung function was found to be due to male sex hormones. So sex differences should be carefully considered when interpreting experimental models of pulmonary fibrosis in mice (Blaauboer et al., 2014). In our study, we used only female mice to avoid the sex differences. We found that anlotinib exerted the preventative effects on bleomycin model of pulmonary fibrosis. Furthermore, anlotinib can also accelerate fibrosis resolution after the establishment of fibrosis.

A recent report showed that anlotinib inhibits the profibrotic effect of TGF-β1 in lung fibroblasts by attenuating inflammation and oxidative stress (Ruan et al., 2020). Our data are in line with that report and reveal an additional mechanism by which anlotinib acts on lung fibroblasts to attenuate fibrosis. There is emerging evidence about the association between metabolic disorders and IPF (Yin et al., 2019; Cho et al., 2020; Hu et al., 2020). Similar to highly proliferative cancer cells, myofibroblasts are highly dependent on glycolysis *in vitro* (Bueno et al., 2020). Furthermore, glycolysis is necessary not only for fibroblast growth and migration but also for the acquisition and maintenance of a myofibroblastic phenotype (Xu et al., 2017; Para et al., 2019). A previous study revealed that inhibition of glycolysis by the PFKFB3 inhibitor 3PO or by genomic disruption of the PFKFB3 gene blunted the differentiation of lung fibroblasts into myofibroblasts and attenuated profibrotic phenotypes in myofibroblasts (Xie et al., 2015). Another study revealed that lung fibroblasts displayed augmented aerobic glycolysis through activation of the PI3K-Akt-mTOR/PFKFB3 pathway in LPS-induced pulmonary fibrosis (Hu et al., 2020). Our data, along with previous studies, demonstrated that glycolytic reprogramming was critical to lung myofibroblast activation and pulmonary fibrosis. Furthermore, we demonstrated that anlotinib could strongly inhibit glycolytic reprogramming *in vitro* and *in vivo*.

The results presented herein provide new insights into the molecular mechanisms of lung fibrogenesis. This work unveils a previously unrecognized posttranscriptional regulation in activated lung fibroblasts composed of the RNA binding protein PCBP3 and the critical glycolytic enzyme PFKFB3, which maintains fibroblasts with higher glycolytic activity in fibrotic lungs compared to normal lung fibroblasts in healthy lungs. PCBP family members perform multiple functions by binding to the poly(C) sequence in both DNA and RNA to modulate mRNA stabilization, translation silencing, or translation enhancement (Blyn et al., 1997; Andino et al., 1999; Ostareck et al., 2001). Our present findings showing that PCBP3 plays an important role in myofibroblast activation and fibrogenesis and significantly extends our previous understanding by identifying an additional node of interaction between PCBP3-mediated posttranscriptional dysregulation and lung disease. We found that PFKFB3 protein overexpression was not accompanied by PFKFB3 mRNA upregulation, indicating that this increase was not transcriptionally derived. Instead, we observed that high PFKFB3 protein levels were maintained during fibroblast transdifferentiation, owing to PCBP3-mediated translational activation. Thus, the PCBP3 protein is upregulated during myofibroblast activation and binds directly to PFKFB3 during transcription. This binding activates PFKFB3 mRNA translation and generates high levels of the glycolysis activator PFKFB3. This mechanism does not exclude additional pathways of regulating PFKFB3 expression. Hence, it is not unusual for key proteins to be regulated at multiple levels, including through transcription, translation, and posttranslational modifications.

Our study is the first to report that anlotinib inhibits PFKFB3-mediated glycolysis in myofibroblasts. Moreover, anlotinib attenuates glycolysis in myofibroblasts by repressing PCBP3 expression levels rather than directly regulating the expression of PFKFB3, as anlotinib treatment does not decrease the mRNA levels of PFKFB3. Our work contributes novel mechanistic insight into the action of anlotinib. However, one of the limitations of this study is that we didn’t knock out PCBP3/PFKFB3 in mice to verify their effects in lung fibrosis, which may be explored in the further research. This future direction may be important to better understand how PCBP3 regulates PFKFB3-mediated glycolysis in pulmonary fibrosis. The other one is that this study only used bleomycin mice model for the research. Although the bleomycin model is the most widely used and best-characterized mouse model, the fibrosis of the bleomycin model is self-resolving, which contrasts with the progressive chronic fibrosis typical of human IPF (Liu et al., 2017). Therefore, whether anlotinib could attenuate fibrosis in human IPF still requires *ex vivo* models of pulmonary fibrosis.

In conclusion, our study demonstrated a clear antifibrotic role for anlotinib in the lungs. Its antifibrotic activity is mediated by its ability to decrease PCBP3 expression and attenuate PFKFB3-driven glycolysis, thereby inhibiting myofibroblast activation. Anlotinib might be considered as a potential therapeutic option for IPF patients.

## Data Availability

The original contributions presented in the study are included in the article/Supplementary Material, further inquiries can be directed to the corresponding authors.

## Abbreviations

IPF, idiopathic pulmonary fibrosis; BLM, bleomycin; RBP, RNA binding protein; α-SMA, α-smooth muscle actin; ECM, extracellular matrix; PFKFB3, 6-phosphofructo-2-kinase/fructose-2, 6-bisphosphatase-3; F2, 6BP, fructose-2, 6-bisphosphate; PFK1, phosphofructokinase-1; TKI, tyrosine kinase inhibitor; VEGFR, vascular endothelial growth factor receptor; EGFR, epidermal growth factor receptor; FGFR, fibroblast growth factor receptor; PDGFR, platelet-derived growth factor receptor; TGF-β1, transforming growth factor-beta 1; TV, tidal volume; Cdyn, dynamic compliance; RI, resistance; HYP, hydroxyproline; IHC, immunohistochemical; H&E, Hematoxylin and eosin; MLF, mouse lung fibroblasts; PPI, protein-protein interaction; MAPK, mitogen-activated protein kinase; ECAR, extracellular acidification rate; siRNA, small interfering RNA; DMSO, dimethylsulfoxide; PBS, phosphate buffered saline; DMEM, Dulbecco’s Modified Eagle Medium; FBS, fetal bovine serum; DAPI, 4′,6-diamidino-2-phenylindole.
