# Supplementary material for: Anlotinib Inhibits PFKFB3-Driven Glycolysis in Myofibroblasts to Reverse Pulmonary Fibrosis
Source: Front Pharmacol. 2021 Sep 16;12:744826. doi: 10.3389/fphar.2021.744826 (PMC8481786; doi:10.3389/fphar.2021.744826)
Supplement: Supplementary file 1 [file DataSheet1.PDF]

Table S1. qPCR primers			
**Gene**	**Species**	**Forward Primer**	**Reverse Primer**
β-actin	Mus musculus	GGCTGTATTCCCCTCCATCG	CCAGTTGGTAACAATGCCATGT
PFKFB3	Mus musculus	CCCAGAGCCGGGTACAGAA	GGGGAGTTGGTCAGCTTCG

SwissTargetPrediction

Target	Common name	Uniprot ID	ChEMBL ID	Target Class	Probability*	Known actives (3D/2D)
Tyrosine-protein kinase receptor UFO	AXL	P30530	CHEMBL4895	Kinase	0.106165761464	72 / 8
Tyrosine-protein kinase receptor TYRO3	TYRO3	Q06418	CHEMBL5314	Kinase	0.106165761464	53 / 3
Proto-oncogene tyrosine-protein kinase MER	MERTK	Q12866	CHEMBL5331	Kinase	0.106165761464	46 / 3
Receptor protein-tyrosine kinase erbB-2	ERBB2	P04626	CHEMBL1824	Kinase	0.106165761464	178 / 5
Tyrosine-protein kinase BRK	PTK6	Q13882	CHEMBL4601	Kinase	0.106165761464	22 / 2
Serine/threonine-protein kinase Aurora-B	AURKB	Q96GD4	CHEMBL2185	Kinase	0.106165761464	143 / 14
Vascular endothelial growth factor receptor 1	FLT1	P17948	CHEMBL1868	Kinase	0.106165761464	79 / 18
Epidermal growth factor receptor erbB1	EGFR	P00533	CHEMBL203	Kinase	0.106165761464	597 / 31
Vascular endothelial growth factor receptor 2	KDR	P35968	CHEMBL279	Kinase	0.106165761464	485 / 95
Tyrosine-protein kinase SRC	SRC	P12931	CHEMBL267	Kinase	0.106165761464	448 / 42
Hepatocyte growth factor receptor	MET	P08581	CHEMBL3717	Kinase	0.106165761464	314 / 108
Serine/threonine-protein kinase GAK	GAK	O14976	CHEMBL4355	Kinase	0.106165761464	16 / 2
Kinesin-1 heavy chain/ Tyrosine-protein kinase receptor RET	RET	P07949	CHEMBL2041	Kinase	0.106165761464	72 / 11
ALK tyrosine kinase receptor	ALK	Q9UM73	CHEMBL4247	Kinase	0.106165761464	207 / 3
Tyrosine-protein kinase ABL	ABL1	P00519	CHEMBL1862	Kinase	0.106165761464	127 / 11
Stem cell growth factor receptor	KIT	P10721	CHEMBL1936	Kinase	0.106165761464	106 / 10
Activin receptor type-1	ACVR1	Q04771	CHEMBL5903	Kinase	0.106165761464	29 / 1
Vascular endothelial growth factor receptor 3	FLT4	P35916	CHEMBL1955	Kinase	0.106165761464	37 / 8
Tyrosine-protein kinase receptor FLT3	FLT3	P36888	CHEMBL1974	Kinase	0.106165761464	156 / 14
Platelet-derived growth factor receptor alpha	PDGFRA	P16234	CHEMBL2007	Kinase	0.106165761464	57 / 14

Target	Common name	Uniprot ID	ChEMBL ID	Target Class	Probability*	Known actives (3D/2D)
Fibroblast growth factor receptor 1	FGFR1	P11362	CHEMBL3650	Kinase	0.106165761464	189 / 9
TGF-beta receptor type I	TGFBR1	P36897	CHEMBL4439	Kinase	0.106165761464	78 / 4
Tyrosine-protein kinase LCK	LCK	P06239	CHEMBL258	Kinase	0.106165761464	167 / 19
Tyrosine-protein kinase BTK	BTK	Q06187	CHEMBL5251	Kinase	0.106165761464	67 / 5
Tyrosine-protein kinase Lyn	LYN	P07948	CHEMBL3905	Kinase	0.106165761464	76 / 6
Fibroblast growth factor receptor 3	FGFR3	P22607	CHEMBL2742	Kinase	0.106165761464	37 / 3
Platelet-derived growth factor receptor beta	PDGFRB	P09619	CHEMBL1913	Kinase	0.106165761464	114 / 8
Tyrosine-protein kinase YES	YES1	P07947	CHEMBL2073	Kinase	0.106165761464	34 / 3
Fibroblast growth factor receptor 2	FGFR2	P21802	CHEMBL4142	Kinase	0.106165761464	35 / 27
Tyrosine-protein kinase FGR	FGR	P09769	CHEMBL4454	Kinase	0.106165761464	17 / 4
Macrophage colony stimulating factor receptor	CSF1R	P07333	CHEMBL1844	Kinase	0.106165761464	149 / 7
Tyrosine-protein kinase BLK	BLK	P51451	CHEMBL2250	Kinase	0.106165761464	20 / 4
Serine/threonine-protein kinase PLK4	PLK4	O00444	CHEMBL3788	Kinase	0.106165761464	14 / 2
Ephrin receptor	EPHB4	P54760	CHEMBL5147	Kinase	0.106165761464	30 / 3
Tyrosine-protein kinase FYN	FYN	P06241	CHEMBL1841	Kinase	0.106165761464	36 / 3
Dual specificity mitogen-activated protein kinase kinase 2	MAP2K2	P36507	CHEMBL2964	Kinase	0.106165761464	11 / 3
Tyrosine-protein kinase HCK	HCK	P08631	CHEMBL3234	Kinase	0.106165761464	30 / 4
Serine/threonine-protein kinase 10	STK10	O94804	CHEMBL3981	Kinase	0.106165761464	13 / 4
Tyrosine-protein kinase ABL2	ABL2	P42684	CHEMBL4014	Kinase	0.106165761464	13 / 4
Tyrosine-protein kinase TIE-2	TEK	Q02763	CHEMBL4128	Kinase	0.106165761464	24 / 16
Ephrin type-A receptor 8	EPHA8	P29322	CHEMBL4134	Kinase	0.106165761464	9 / 3
Serine/threonine-protein kinase 2	SLK	Q9H2G2	CHEMBL4202	Kinase	0.106165761464	17 / 4
Tyrosine-protein kinase FRK	FRK	P42685	CHEMBL4223	Kinase	0.106165761464	15 / 3
Ephrin type-A receptor 6	EPHA6	Q9UF33	CHEMBL4526	Kinase	0.106165761464	10 / 4
TRAF2- and NCK-	TNIK	Q9UKE5	CHEMBL4527	Kinase	0.106165761464	20 / 4

Target	Common name	Uniprot ID	ChEMBL ID	Target Class	Probability*	Known actives (3D/2D)
interacting kinase						
Serine/threonine-protein kinase MST1	STK4	Q13043	CHEMBL4598	Kinase	0.106165761464	11 / 3
Mitogen-activated protein kinase kinase kinase kinase 5	MAP4K5	Q9Y4K4	CHEMBL4852	Kinase	0.106165761464	12 / 4
Casein kinase I epsilon	CSNK1E	P49674	CHEMBL4937	Kinase	0.106165761464	12 / 3
Ephrin type-A receptor 3	EPHA3	P29320	CHEMBL4954	Kinase	0.106165761464	11 / 3
Tyrosine-protein kinase receptor Tie-1	TIE1	P35590	CHEMBL5274	Kinase	0.106165761464	11 / 4
Mitogen-activated protein kinase kinase kinase kinase 3	MAP4K3	Q8IVH8	CHEMBL5432	Kinase	0.106165761464	14 / 3
Serine/threonine-protein kinase SIK2	SIK2	Q9H0K1	CHEMBL5699	Kinase	0.106165761464	12 / 3
Mitogen-activated protein kinase kinase kinase kinase 1	MAP4K1	Q92918	CHEMBL5749	Kinase	0.106165761464	14 / 3
Serine/threonine-protein kinase 33	STK33	Q9BYT3	CHEMBL6005	Kinase	0.106165761464	16 / 4
Mitogen-activated protein kinase kinase kinase kinase 4	MAP4K4	O95819	CHEMBL6166	Kinase	0.106165761464	17 / 4
Serine/threonine-protein kinase TAO2	TAOK2	Q9UL54	CHEMBL1075195	Kinase	0.106165761464	7 / 3
Serine/threonine-protein kinase TAO3	TAOK3	Q9H2K8	CHEMBL5701	Kinase	0.106165761464	12 / 2
Voltage-gated calcium channel alpha2/delta subunit 1	CACNA2D1	P54289	CHEMBL1919	Calcium channel auxiliary subunit alpha2delta family	0.106165761464	15 / 0
Voltage-gated calcium channel alpha2/delta subunit 2	CACNA2D2	Q9NY47	CHEMBL3896	Calcium channel auxiliary subunit alpha2delta family	0.106165761464	4 / 0
Receptor protein-tyrosine kinase erbB-4	ERBB4	Q15303	CHEMBL3009	Kinase	0.106165761464	29 / 3
Mitogen-activated protein kinase kinase kinase 8	MAP3K8	P41279	CHEMBL4899	Kinase	0.106165761464	32 / 0
Serine/threonine-protein kinase/endoribonuclease IRE1	ERN1	O75460	CHEMBL1163101	Enzyme	0.106165761464	18 / 1
Macrophage-	MST1R	Q04912	CHEMBL2689	Kinase	0.106165761464	8 / 11

Target	Common name	Uniprot ID	ChEMBL ID	Target Class	Probability*	Known actives (3D/2D)
stimulating protein receptor						
Serine/threonine-protein kinase Aurora-C	AURKC	Q9UQB9	CHEMBL3935	Kinase	0.106165761464	15 / 2
Fibroblast growth factor receptor 4	FGFR4	P22455	CHEMBL3973	Kinase	0.106165761464	15 / 2
Ephrin type-A receptor 7	EPHA7	Q15375	CHEMBL4602	Kinase	0.106165761464	7 / 3
Dual specificity mitogen-activated protein kinase kinase 5	MAP2K5	Q13163	CHEMBL4948	Kinase	0.106165761464	11 / 4
Serine/threonine-protein kinase RIPK2	RIPK2	O43353	CHEMBL5014	Kinase	0.106165761464	6 / 4
Discoidin domain-containing receptor 2	DDR2	Q16832	CHEMBL5122	Kinase	0.106165761464	13 / 3
Activin receptor type-1B	ACVR1B	P36896	CHEMBL5310	Kinase	0.106165761464	6 / 1
Epithelial discoidin domain-containing receptor 1	DDR1	Q08345	CHEMBL5319	Kinase	0.106165761464	14 / 4
Mitogen-activated protein kinase kinase kinase kinase 2	MAP4K2	Q12851	CHEMBL5330	Kinase	0.106165761464	15 / 3
Misshapen-like kinase 1	MINK1	Q8N4C8	CHEMBL5518	Kinase	0.106165761464	14 / 4
Leukocyte tyrosine kinase receptor	LTK	P29376	CHEMBL5627	Kinase	0.106165761464	10 / 3
Serine/threonine-protein kinase 35	STK35	Q8TDR2	CHEMBL5651	Kinase	0.106165761464	9 / 4
Ephrin type-A receptor 1	EPHA1	P21709	CHEMBL5810	Kinase	0.106165761464	9 / 3
Ephrin receptor	EPHB6	O15197	CHEMBL5836	Unclassified protein	0.106165761464	11 / 4
Receptor tyrosine-protein kinase erbB-3	ERBB3	P21860	CHEMBL5838	Kinase	0.106165761464	4 / 3
Serine/threonine-protein kinase MST4	STK26	Q9P289	CHEMBL5941	Kinase	0.106165761464	12 / 2
Eukaryotic translation initiation factor 2-alpha kinase 1	EIF2AK1	Q9BQI3	CHEMBL6029	Kinase	0.106165761464	3 / 3
SPS1/STE20-related protein kinase YSK4	MAP3K19	Q56UN5	CHEMBL6191	Kinase	0.106165761464	15 / 4
Serine/threonine-protein kinase AKT2	AKT2	P31751	CHEMBL2431	Kinase	0.106165761464	318 / 0
Protein kinase C gamma	PRKCG	P05129	CHEMBL2938	Kinase	0.106165761464	38 / 0

Target	Common name	Uniprot ID	ChEMBL ID	Target Class	Probability*	Known actives (3D/2D)
Serine/threonine-protein kinase AKT	AKT3	Q9Y243	CHEMBL4816	Kinase	0.106165761464	73 / 0
Serine/threonine-protein kinase PIM1	PIM1	P11309	CHEMBL2147	Kinase	0.106165761464	638 / 0
Serine/threonine-protein kinase PIM2	PIM2	Q9P1W9	CHEMBL4523	Kinase	0.106165761464	448 / 0
Serine/threonine-protein kinase PIM3	PIM3	Q86V86	CHEMBL5407	Kinase	0.106165761464	331 / 0
Cyclin-dependent kinase 2/cyclin E1	CCNE1 CDK2	P24864 P24941	CHEMBL1907605	Kinase	0.106165761464	74 / 0
Tyrosine-protein kinase JAK1	JAK1	P23458	CHEMBL2835	Kinase	0.106165761464	137 / 0
Dipeptidyl peptidase VIII	DPP8	Q6V1X1	CHEMBL4657	Protease	0.106165761464	346 / 0
Dipeptidyl peptidase IX	DPP9	Q86TI2	CHEMBL4793	Protease	0.106165761464	239 / 0
Phosphodiesterase 4B	PDE4B	Q07343	CHEMBL275	Phosphodiesterase	0.106165761464	43 / 0
Protein kinase C iota	PRKCI	P41743	CHEMBL2598	Kinase	0.106165761464	287 / 0
Cyclin-dependent kinase 2	CDK2	P24941	CHEMBL301	Kinase	0.106165761464	170 / 0
Cyclin-dependent kinase 1	CDK1	P06493	CHEMBL308	Kinase	0.106165761464	146 / 0
Cyclin T1	CCNT1	O60563	CHEMBL2108	Other cytosolic protein	0.106165761464	111 / 0
Telomerase reverse transcriptase	TERT	O14746	CHEMBL2916	Enzyme	0.106165761464	79 / 0
Sodium/calcium exchanger 1	SLC8A1	P32418	CHEMBL4076	Electrochemical transporter	0.106165761464	44 / 0
Gonadotropin-releasing hormone receptor	GNRHR	P30968	CHEMBL1855	Family A G protein-coupled receptor	0.106165761464	431 / 0
Amine oxidase, copper containing	AOC3	Q16853	CHEMBL3437	Enzyme	0.106165761464	19 / 0

Table S3. GeneCards							
Number	Gene	Number	Gene	Number	Gene	Number	Gene
1	CFTR	335	PDGFA	669	CPLANE1	1003	HSPH1
2	TGFB1	336	MIR197	670	MKI67	1004	MT-ATP6
3	TERT	337	MIRLET7B	671	TBX1	1005	MIR212
4	TP53	338	MIR195	672	PI3	1006	CASP10
5	TNF	339	MIR96	673	BMP4	1007	PDE5A
6	SFTPC	340	CHI3L1	674	PMS2	1008	SH2D1A
7	EGFR	341	MIRLET7E	675	SLC7A7	1009	SCN5A
8	IL6	342	MIR16-1	676	E2F1	1010	RSPH4A
9	MUC5B	343	MIR199A1	677	S100A1	1011	PIK3R2
10	RTEL1	344	MIR210	678	TKT	1012	DNAH9
11	IL10	345	MIR20A	679	MYRF	1013	PSMA7
12	SFTPB	346	MIR378A	680	LAMA5	1014	KIF3A
13	SFTPA1	347	THBD	681	COL2A1	1015	CYP3A5
14	ELN	348	DMBT1	682	DEFB1	1016	LOC111674466
15	ABCA3	349	MIR185	683	RPL5	1017	ADM
16	CAV1	350	IL9	684	SOD1	1018	PSMC3
17	IFNG	351	CFM1	685	PLK1	1019	WDPCP
18	MUC1	352	CD79A	686	SCGB3A2	1020	MIR101-1
19	SFTPA2	353	DYNC2LI1	687	SLC9A3R1	1021	PRKCB
20	SERPINA1	354	NEK1	688	IREB2	1022	IL13RA2
21	STAT3	355	IQCB1	689	LOC113664106	1023	MYH11
22	TERC	356	MIR204	690	FADD	1024	PRKG2
23	CCN2	357	HLA-A	691	CYP3A4	1025	SUFU
24	IL13	358	IGF2	692	HSPB1	1026	PIK3C2A
25	TLR4	359	FLNA	693	MAPK14	1027	PSMC5
26	PARN	360	BCL2	694	CTSB	1028	KCNQ1OT1
27	CTNNB1	361	GSN	695	IGF2R	1029	MIR133A1
28	CCR6	362	MAP2K2	696	BAP1	1030	GOPC
29	AKT1	363	BPIFA1	697	GATA2	1031	TRAF2
30	MIR21	364	MIR15A	698	MMP8	1032	TNFRSF6B
31	NKX2-1	365	EPHX1	699	ITGAV	1033	MIR139
32	MMP1	366	HPS5	700	PTX3	1034	LOC111674470
33	SMAD4	367	TRAF3IP1	701	MSLN	1035	IGHMBP2
34	ACE	368	CFTR-AS1	702	AFF4	1036	PSMA3
35	SPP1	369	AP3B1	703	CTCF	1037	PSMC6
36	CXCL8	370	GAPDH	704	ADA	1038	GADD45B
37	EGF	371	KRT19	705	KITLG	1039	FKRP
38	FASLG	372	MIR107	706	ZNF423	1040	DNAAF2
39	CDH1	373	CP	707	IL12A	1041	ERCC5
40	KRAS	374	HLA-B	708	PRKCA	1042	E2F3
41	SERPINE1	375	MMP12	709	EGR1	1043	PIK3R3
42	BRAF	376	AGER	710	DLL4	1044	PRF1
43	SMAD3	377	DDR1	711	IFNA1	1045	IFNAR1
44	PRTN3	378	TNNT2	712	EZR	1046	PF4
45	PIK3CA	379	CCL18	713	LGALS3	1047	MIR27B
46	IL1RN	380	MIR148B	714	AFP	1048	VTN
47	HMOX1	381	MIR141	715	TUBB2B	1049	DNAJC5
48	IL1B	382	INS	716	EPCAM	1050	BGLAP
49	HLA-DRB1	383	GLI3	717	APOB	1051	MIR455
50	FAM13A	384	JAK2	718	SPPL2C	1052	LOC111674471
51	FAS	385	EVC	719	CDK2	1053	CSF3R
52	PTEN	386	HPS3	720	GDF2	1054	VCL

53	SFTPD	387	IL33	721	EPO	1055	NAGLU
54	ITGAM	388	NOS2	722	VCP	1056	GUSB
55	DSP	389	SLPI	723	HSPA8	1057	NEAT1
56	STN1	390	MIR133B	724	ATP4A	1058	TBX4
57	TGFBR1	391	TCTN3	725	HBB	1059	PSMD1
58	SRC	392	TLR5	726	SELE	1060	PSMD12
59	CDKN2A	393	MUC4	727	NAT2	1061	GAST
60	ERBB2	394	MIR93	728	FGF10	1062	MIR99A
61	CTLA4	395	MIR18A	729	BMP7	1063	CREB1
62	NFE2L2	396	SLC11A1	730	ETS1	1064	TRPC6
63	MMP9	397	PSMA6	731	DEFB4A	1065	EPAS1
64	MTOR	398	RNASE3	732	PRDM10	1066	MIR125B1
65	HRAS	399	SMAD2	733	GJA1	1067	IFNGR1
66	TINF2	400	ICAM1	734	RPS6KB1	1068	PGR
67	FGFR1	401	TGFA	735	JAK3	1069	TLR1
68	COL1A1	402	RPGR	736	TP63	1070	CD40
69	NPHP3	403	ANXA5	737	HNF1B	1071	DLK1
70	F2	404	CXCR1	738	BBS10	1072	FOS
71	DPP9	405	IL2	739	ITGA2	1073	DNAL1
72	TGFB2	406	GREM1	740	HOTAIR	1074	MAD1L1
73	ATP11A	407	CD274	741	SGK1	1075	PSMD11
74	HFE	408	MIR142	742	DNAAF1	1076	HBA2
75	PDGFRB	409	GSTP1	743	DNMT1	1077	RAB11B
76	ITGA3	410	PHOX2A	744	DAB2	1078	CCKBR
77	MIRLET7D	411	SOS2	745	CDH2	1079	CLCN3
78	MBL2	412	LMNA	746	HJV	1080	PPARA
79	NOTCH1	413	ENPP2	747	PRKAA1	1081	MB
80	MET	414	TOP1	748	HSPA5	1082	GAS5-AS1
81	TIMP1	415	APEX1	749	CHUK	1083	LOC111674474
82	SERPINH1	416	CXCL9	750	SKP2	1084	KCNJ1
83	SCNN1A	417	PLAU	751	ILK	1085	KIAA0319L
84	MMP2	418	MIRLET7C	752	ADORA2B	1086	PSMA2
85	ELANE	419	MIR30E	753	SST	1087	PSMD3
86	VEGFA	420	MIR214	754	CFLAR	1088	LOC111674473
87	PLG	421	MIR486-1	755	CCNB1	1089	ABCF2
88	MIR130A	422	KRT7	756	ADCY10	1090	PSMD8
89	ADRB2	423	TNFRSF13B	757	HDAC9	1091	MIR590
90	KIT	424	MIR106B	758	ARG1	1092	PSMD7
91	CC2D2A	425	MIR181A1	759	BAK1	1093	RNF5
92	TGFBR2	426	MTHFR	760	DICER1	1094	GLIS3
93	PDGFRA	427	BTNL2	761	IKBKG	1095	CHAT
94	IL4	428	PTPN11	762	MYH6	1096	LOC111674476
95	NHP2	429	KDR	763	COL4A3	1097	MIR23B
96	STK11	430	ELMOD2	764	FGA	1098	MIR130B
97	NOP10	431	STING1	765	ABL1	1099	MIR151A
98	ALOX5	432	THY1	766	GJA5	1100	LOC111674478
99	TMEM67	433	MIR140	767	ARHGAP31	1101	CD82
100	CCL2	434	VCAM1	768	FCGR3B	1102	E2F2
101	MIR34C	435	CCR2	769	SLC26A9	1103	RNF185
102	STAT1	436	CDKN1A	770	LRBA	1104	MIR423
103	ALB	437	STAT4	771	PMM2	1105	DEFB103B
104	HGF	438	CEP164	772	ACD	1106	NFKB2
105	RPGRIP1L	439	MIR128-2	773	CHIT1	1107	MIR186
106	NPHP1	440	S100A9	774	CDK6	1108	AFAP1-AS1

107	TSC2	441	TLR3	775	CSF1	1109	PSMC2
108	MDM2	442	SERPINA3	776	ABCG2	1110	ERLIN2
109	TTC21B	443	MIR25	777	CASP1	1111	GADD45G
110	EDN1	444	CD44	778	P2RX7	1112	CS
111	WDR19	445	MALAT1	779	ASCL1	1113	CD22
112	CRP	446	COL3A1	780	VWF	1114	PSMB1
113	CCL11	447	CD28	781	PROM1	1115	LOC111674479
114	AGT	448	MIR23A	782	PDPN	1116	LOC111674467
115	CCND1	449	MIR19A	783	PRODH	1117	SDHB
116	COL1A2	450	HAMP	784	ALG9	1118	LOC111674465
117	COPA	451	MIR429	785	HOXD13	1119	PSMD14
118	CASP8	452	MIR335	786	TNFRSF10A	1120	IFNB1
119	DKC1	453	TTR	787	IL12B	1121	PLAT
120	ACTC1	454	C2CD3	788	BBS9	1122	PSMA5
121	FGFR2	455	NR1H4	789	NR3C1	1123	NEB
122	IL17A	456	G6PD	790	MIR122	1124	CHGA
123	SCNN1B	457	MIR127	791	ITGB3	1125	MIR187
124	ATM	458	TNNI3	792	ERF	1126	LOC113523647
125	MIR155	459	CXCL1	793	CCR5	1127	HSD3B7
126	SHH	460	MIR424	794	ADIPOQ	1128	LOC108491823
127	RB1	461	IFT52	795	TF	1129	IL4R
128	CSF2	462	ZCCHC8	796	RXRA	1130	PSMB4
129	CEP290	463	AGTR2	797	FAT4	1131	DRC1
130	FCGR2A	464	NSMCE3	798	PSMD4	1132	MIR361
131	PKHD1	465	TYR	799	UMOD	1133	COL11A2
132	NPHP4	466	ABCB4	800	TMEM107	1134	MIR497
133	MIR200B	467	NOS3	801	BLOC1S6	1135	SBDS
134	MKS1	468	LPAR1	802	MMP14	1136	TLR6
135	SCNN1G	469	CXCR2	803	PTK2B	1137	MIR339
136	FAM111B	470	ZIC3	804	PIEZO2	1138	RECQL4
137	FN1	471	LOC111674475	805	CDK1	1139	DERL2
138	INVS	472	CCR7	806	SIRT3	1140	SH2B3
139	STAT6	473	ANKS6	807	IL15	1141	GUCA2A
140	WRAP53	474	CALR	808	TAP1	1142	CCDC103
141	FGFR3	475	TCTN1	809	GATA6	1143	PCNA
142	RET	476	MIR224	810	EPOR	1144	PSME2
143	NRAS	477	IGF1R	811	PVT1	1145	NCOR1
144	DDR2	478	XRCC1	812	MUSK	1146	TTC37
145	FBN1	479	CD34	813	ESR2	1147	PSMB5
146	OFD1	480	EVC2	814	MAP1B	1148	PRKG1
147	MAP2K1	481	CEACAM5	815	MT-CYB	1149	LEPR
148	ENG	482	MIR338	816	TRAF6	1150	TNC
149	NEK8	483	GATA4	817	PKD1L1	1151	PTGS1
150	FGF2	484	MIRLET7G	818	RBPJ	1152	IFNA2
151	MUC5AC	485	SOD2	819	DMD	1153	DZIP1L
152	NFKB1	486	MIR191	820	FABP4	1154	MIR296
153	RARB	487	CCR3	821	CD80	1155	CD86
154	TNFRSF1A	488	CXCL5	822	PSMA4	1156	MYCL
155	IL5	489	MYLK	823	IGHE	1157	VPS45
156	APC	490	H2AC18	824	EGFR-AS1	1158	GBE1
157	SMPD1	491	GPC3	825	DNMT3B	1159	IL7R
158	MPO	492	TMEM138	826	MIR26A1	1160	CYP27A1
159	ACTA2	493	MIR377	827	FLT4	1161	PLA2G7
160	MIR126	494	CCL22	828	CCNA2	1162	NOTCH2

161	NOD2	495	SLC34A2	829	DNAI2	1163	CEACAM1
162	MIR17	496	PTPN22	830	TUG1	1164	MIR485
163	FGF7	497	PKD2	831	IFIH1	1165	LOC113633876
164	IFT140	498	DNAH5	832	KATNIP	1166	REG3A
165	KRT18	499	S100A4	833	MMP3	1167	EPRS1
166	REN	500	BAX	834	KLF6	1168	LOC113633875
167	PDCD1	501	THPO	835	BLOC1S3	1169	LOC113604967
168	VEGFC	502	MIR137	836	SERPINB1	1170	ZEB1
169	IFT80	503	FOXJ1	837	GLA	1171	FOXM1
170	SCGB1A1	504	BCL2L1	838	LPO	1172	CASR
171	BRCA2	505	TRPV4	839	F2R	1173	TALDO1
172	AHI1	506	CCR4	840	BTK	1174	MIR219A1
173	TCTN2	507	CADM1	841	SOCS3	1175	CCL26
174	BMPR2	508	TMEM237	842	COL4A2	1176	LOC113664107
175	RAF1	509	LOC111674463	843	NBAS	1177	CLCN4
176	CXCR4	510	GBA	844	CEP104	1178	SNHG1
177	KIAA0586	511	EZH2	845	MAGEA4	1179	LAMA2
178	HIF1A	512	SOX9	846	DERL1	1180	TCF7
179	FARSB	513	MIR24-2	847	AKT3	1181	TFRC
180	TLR2	514	SPARC	848	NPPA	1182	PLCZ1
181	AGTR1	515	S100A8	849	BCL2L11	1183	POMC
182	BMP6	516	LTA	850	LAMA1	1184	ABCC3
183	CDK4	517	MKKS	851	IL3	1185	CRYAB
184	TMEM216	518	HYDIN	852	ITGA5	1186	LOC110806263
185	RELA	519	GLI1	853	CYP2D6	1187	MCM4
186	CSF3	520	BIRC5	854	DOCK6	1188	TNFSF13B
187	CCL5	521	MAPK3	855	CANX	1189	MIR149
188	ESR1	522	PSMB8	856	AXIN2	1190	PLA2G6
189	MIR223	523	AP3D1	857	SCARB2	1191	KNG1
190	FOXP3	524	MIRLET7A3	858	CDX2	1192	HOTTIP
191	TTN	525	IDH1	859	SLC9A3	1193	AR
192	MARS1	526	CYP2E1	860	ICOS	1194	PSMB2
193	MAPK8	527	TNFSF10	861	PTGER4	1195	LSM1
194	MEG3	528	PRKCD	862	SLC17A5	1196	NIPBL
195	CCL3	529	MIR181C	863	CXCR5	1197	PSMA8
196	HPS4	530	CYCS	864	TCTEX1D2	1198	IL16
197	BRCA1	531	IFT27	865	MXRA5	1199	CCAT1
198	IL2RA	532	TUBB3	866	FOXP1	1200	SPRY2
199	HPS1	533	HP	867	MIR138-1	1201	RIPK1
200	MIR34A	534	ALMS1	868	CLDN1	1202	PHKG2
201	MIR200C	535	NOX4	869	MIR154	1203	MIR503
202	B9D1	536	RYR1	870	INSR	1204	GSTT1
203	DNASE1	537	TSLP	871	UCHL1	1205	PRKCI
204	TSC1	538	VDAC1	872	DCN	1206	AGL
205	WDR35	539	U2AF1	873	XPNPEP3	1207	RAG2
206	WT1	540	CAT	874	KRT13	1208	HNF1A-AS1
207	INPP5E	541	CALCA	875	COMT	1209	CCAT2
208	MPL	542	DNAH11	876	EDNRA	1210	ERLIN1
209	MIR125A	543	NF1	877	CA4	1211	MIR125B2
210	CSPP1	544	PTK2	878	HLA-DQA1	1212	MIR15B
211	GRP	545	SP110	879	BBS12	1213	CREBBP
212	MIR144	546	IL12RB1	880	KRT8	1214	CTSL
213	RTEL1-TNFRSF6B	547	NQO1	881	CMA1	1215	NR5A1
214	SERPINC1	548	GAA	882	AKR1B10	1216	MSR1

215	CD4	549	MIR409	883	LOC113633877	1217	UTP4
216	DYNC2H1	550	VIP	884	CEP55	1218	MIR301A
217	MIR145	551	GPT	885	MAP2K4	1219	MUC7
218	FLT1	552	MIR708	886	CDK5	1220	CASC2
219	TNFRSF1B	553	NFKBIA	887	P2RY2	1221	ZNRD1ASP
220	MIR29A	554	KEAP1	888	NLRP3	1222	PLCG2
221	HLA-DQB1	555	LOC111674477	889	PSMC4	1223	MIR124-1
222	FHIT	556	CD36	890	HSPA4	1224	MIR382
223	H19	557	IGFBP3	891	AVPR2	1225	LAMP1
224	PKD1	558	NKX2-5	892	RPS27A	1226	CD69
225	SOS1	559	CEP83	893	MIR375	1227	DANCR
226	SPINK1	560	BDNF	894	MIR33A	1228	TP53COR1
227	MIR31	561	ABCB11	895	ATF6	1229	MYL3
228	IFT172	562	FGFR4	896	RXRB	1230	CYSLTR2
229	MMP7	563	CTSG	897	UBC	1231	SELL
230	CD8A	564	ITGB1	898	NPC2	1232	LINC-ROR
231	PDGFB	565	CSF2RA	899	CLCN2	1233	NOS1
232	SLC2A1	566	DNAI1	900	FOXE1	1234	IFT20
233	JUN	567	ARL3	901	MIR132	1235	MIR10B
234	BMP2	568	MECP2	902	HDGF	1236	CD81
235	HMGB1	569	CDKN1B	903	WNT7B	1237	ANTXR1
236	ERBB3	570	DNMT3A	904	SLC9A3R2	1238	PRSS2
237	MIR150	571	VHL	905	H2AX	1239	SNHG20
238	PRSS1	572	THBS1	906	PRKAG2	1240	MIR95
239	MIR29C	573	YAP1	907	SERPINB3	1241	GALC
240	MIR146B	574	TIMP2	908	LRP1B	1242	DGCR5
241	ABCC1	575	TOLLIP	909	DNAAF3	1243	HNMT
242	BBS2	576	KCNK3	910	BCL10	1244	SLCO2A1
243	ABCB1	577	NOTCH3	911	RAG1	1245	MLH1
244	CXCL10	578	RASSF1	912	LAMA4	1246	PLA2G2A
245	XIAP	579	SP1	913	HLA-G	1247	MME
246	KIF21A	580	RHOA	914	PSMB3	1248	TYMS
247	PIK3R1	581	ABCC2	915	SIRT1	1249	MIR198
248	BBS1	582	IKBKB	916	MIRLET7A1	1250	JAK1
249	IRF1	583	GZMB	917	PLA2G1B	1251	PDE4D
250	IL1A	584	LEP	918	KDM4C	1252	LAMC2
251	PTPRC	585	BBS5	919	MUC6	1253	AHR
252	HLA-DPB1	586	APOE	920	TUBB1	1254	TPM1
253	ERCC6	587	NHLRC2	921	SKIV2L	1255	MT-CO2
254	STX1A	588	NPPB	922	TPM2	1256	ASCC1
255	SETD2	589	SYP	923	CCDC40	1257	EOGT
256	CCL17	590	ACTB	924	TET2	1258	CCL7
257	IDH2	591	PSMA1	925	TRIP11	1259	TRPM4
258	CXCL12	592	PTCH1	926	ITGA2B	1260	GPSM2
259	MIR27A	593	LOXL2	927	ENO2	1261	TARS1
260	GSTM1	594	CEP41	928	EIF2AK3	1262	TPM3
261	DCTN4	595	MYPN	929	RSPH9	1263	CD14
262	PRKN	596	CR1	930	MIR193A	1264	MIR216A
263	CXCR3	597	ERCC1	931	PXN	1265	CTAG1B
264	MIR200A	598	IGFBP5	932	DNAAF5	1266	MIR22HG
265	ARL13B	599	MGMT	933	LAT	1267	MIR10A
266	PTGS2	600	VIM	934	RSPH1	1268	PCAT1
267	MIR148A	601	ENO1	935	CDKN2B-AS1	1269	SNHG15
268	MYC	602	NLRC4	936	ADORA1	1270	BANCR

269	IGF1	603	PIK3CG	937	RAD51	1271	PSAP
270	IL18	604	MYH7	938	DIABLO	1272	IL11
271	CYP1A1	605	F13A1	939	TFR2	1273	MIR362
272	NEK9	606	WNT4	940	RSPO2	1274	UCA1
273	LOX	607	NME1	941	C4A	1275	UBE2L3
274	MIR30D	608	CD63	942	IFI27	1276	FBLN5
275	MIR146A	609	RPGRIP1	943	MIR29B1	1277	GC
276	IFT122	610	ATP8B1	944	HSPD1	1278	TP73-AS1
277	B9D2	611	GNAS	945	CCDC39	1279	PPBP
278	KCNQ1	612	TUBB	946	PDE4A	1280	LINC00473
279	CYP2A6	613	IFRD1	947	TRIM21	1281	SOX2-OT
280	MIR483	614	CASP9	948	PLCG1	1282	MIR181B1
281	MIR183	615	LAMP2	949	IL1RL1	1283	XIST
282	IL1R1	616	HDAC2	950	BBIP1	1284	MIR129-1
283	SDCCAG8	617	ANXA1	951	MIR196A1	1285	PRL
284	PTRH2	618	IFT74	952	BAD	1286	MIR193B
285	MIR182	619	ROS1	953	MYBPC3	1287	NR3C2
286	CDKN3	620	CLEC7A	954	ARAF	1288	MYL1
287	GUCY2C	621	AKT2	955	VDR	1289	SDC1
288	MIR222	622	WRN	956	MAGEA1	1290	MIR24-1
289	LOC111674472	623	DTNBP1	957	TAC1	1291	PHB
290	KIF7	624	CD19	958	IDUA	1292	MYL2
291	FLNC	625	SMARCA4	959	EPX	1293	APOA1
292	PPARG	626	ANGPT2	960	ASXL1	1294	SNHG12
293	XRCC3	627	PARP1	961	MAGEA3	1295	CEP57
294	SNAI1	628	BBS7	962	RAC1	1296	IL6R
295	POSTN	629	MIF	963	GATA1	1297	SERPINF2
296	MIR192	630	SELP	964	GAS5	1298	CALB2
297	JAG1	631	HSPG2	965	MIR9-1	1299	MIR152
298	CD40LG	632	ATP12A	966	RYR2	1300	ADK
299	DYNC2I1	633	HSP90AA1	967	JPH2	1301	NRG1
300	ACVRL1	634	CHRM3	968	RIOX2	1302	MIR501
301	MIR221	635	MVP	969	LOC111674464	1303	GPRC5A
302	IFT43	636	DCDC2	970	TNFAIP3	1304	LZTR1
303	DES	637	ERCC2	971	PIK3CB	1305	TLR7
304	MIR22	638	PPP2R1B	972	DHCR7	1306	POT1
305	TNFRSF10B	639	TYMP	973	C1S	1307	EP300
306	IRF5	640	CLCA1	974	SNAI2	1308	FBL
307	ALK	641	OGG1	975	ERBB4	1309	PLOD2
308	CEP120	642	MCL1	976	ACVR1	1310	DLL1
309	MIR451A	643	CCN4	977	NPC1	1311	TOP2A
310	DYNC2I2	644	F3	978	LNX1	1312	MAGEC2
311	CASP3	645	STAT5B	979	GRB2	1313	MIR499A
312	CCL4	646	FUZ	980	TFAP2B	1314	ENSG00000266919
313	HPS6	647	LBR	981	SLC6A4	1315	TNFRSF11B
314	MIR143	648	CXCL2	982	IFT88	1316	HOXA11-AS
315	ICOSLG	649	PLAUR	983	NPHS1	1317	RASGRP1
316	CLCA4	650	ASAH1	984	MAP2K7	1318	SPRY4-IT1
317	HLA-DPA1	651	BPI	985	PSMD2	1319	FIP1L1
318	FOXF1	652	NPM1	986	DNAH8	1320	GSR
319	BIRC3	653	ACTA1	987	B2M	1321	CST3
320	SMAD7	654	ANGPT1	988	INTU	1322	LTBP4
321	MIR203A	655	GGT1	989	ENTPD1	1323	ZFAS1
322	MIR30A	656	CAMP	990	PRSS8	1324	F5

323	MIR324	657	SOD3	991	CCNE1	1325	STMN1
324	MIR199B	658	F2RL3	992	LRRC56	1326	AIRE
325	ACP5	659	TEK	993	MAP3K8	1327	RETN
326	MIR205	660	LTF	994	SHC1	1328	NTS
327	TP73	661	MT-CO1	995	CCDC114	1329	KRT5
328	TLR9	662	LIPA	996	ACHE	1330	F2RL1
329	MAPK1	663	WNT3	997	TBX20	1331	TNFSF11
330	CLCN5	664	GDF1	998	AURKB	1332	COL4A5
331	TMEM231	665	SLC40A1	999	ASL	1333	PIK3CD
332	BBS4	666	GLIS2	1000	MIR30C1	1334	FGF9
333	RMRP	667	AREG	1001	IL2RB	1335	KRT20
334	TGFB3	668	CDKN2B	1002	NCF2		

Table S4. Common targets			
**Number**	**Gene**	**Number**	**Gene**
1	AXL	38	EPHA3
2	MERTK	39	TIE1
3	ERBB2	40	CACNA2D1
4	AURKB	41	ERBB4
5	FLT1	42	MAP3K8
6	EGFR	43	ERN1
7	KDR	44	MST1R
8	SRC	45	FGFR4
9	MET	46	EPHA7
10	RET	47	MAP2K5
11	ALK	48	RIPK2
12	ABL1	49	DDR2
13	KIT	50	ACVR1B
14	ACVR1	51	DDR1
15	FLT4	52	MAP4K2
16	FLT3	53	EPHA1
17	PDGFRA	54	EPHB6
18	FGFR1	55	ERBB3
19	TGFBR1	56	EIF2AK1
20	BTK	57	MAP3K19
21	LYN	58	AKT2
22	FGFR3	59	PRKCG
23	PDGFRB	60	AKT3
24	YES1	61	PIM1
25	FGFR2	62	PIM2
26	FGR	63	JAK1
27	CSF1R	64	DPP8
28	BLK	65	DPP9
29	PLK4	66	PDE4B
30	FYN	67	PRKCI
31	MAP2K2	68	CDK2
32	HCK	69	CDK1
33	STK10	70	CCNT1
34	ABL2	71	TERT
35	TEK	72	SLC8A1
36	SLK	73	GNRHR
37	STK4	74	AOC3

Table S5. Potential proteins may interact with PFKFB3 mRNA through the catRAPID algorithm.

#	Protein ID	RNA ID	Z-score?	Discriminative Power (%)?	Interaction Strength (%)?	Domain?	Motif?	Ranking?
1	ELAV1_MOUSE_247-308	NC_000068.7:c11_1_5924-6036	-0.13	50	98	yes	yes	★★★
2	ELAV1_MOUSE_247-308	NC_000068.7:c11_1_26816-27008	0.08	67	99	yes	yes	★★★
3	ELAV1_MOUSE_247-308	NC_000068.7:c11_1_15806-15983	-0.23	40	90	yes	yes	★★★
4	PCBP3_MOUSE_301-351	NC_000068.7:c11_1_41382-41564	-0.69	14	13	yes	yes	★★★
5	ELAV1_MOUSE_247-308	NC_000068.7:c11_1_77223-77340	-0.47	22	74	yes	yes	★★★
6	ELAV1_MOUSE_247-308	NC_000068.7:c11_1_10399-10532	-0.50	20	64	yes	yes	★★★
7	ELAV1_MOUSE_109-176	NC_000068.7:c11_1_5924-6036	-0.14	50	98	yes	yes	★★★
8	ELAV1_MOUSE_109-176	NC_000068.7:c11_1_45706-45815	-0.39	26	85	yes	yes	★★★
9	ELAV1_MOUSE_109-176	NC_000068.7:c11_1_26816-27008	0.04	63	99	yes	yes	★★★
10	ELAV1_MOUSE_109-176	NC_000068.7:c11_1_15806-15983	-0.24	40	90	yes	yes	★★★
11	ELAV1_MOUSE_109-172	NC_000068.7:c11_1_5924-6036	-0.15	47	97	yes	yes	★★★
12	ELAV1_MOUSE_109-172	NC_000068.7:c11_1_45706-45815	-0.40	26	85	yes	yes	★★★
13	ELAV1_MOUSE_109-172	NC_000068.7:c11_1_15806-15983	-0.26	37	87	yes	yes	★★★
14	PCBP3_MOUSE_301-351	NC_000068.7:c11_1_77461-77636	-0.71	14	9	yes	yes	★★★
15	PCBP3_MOUSE_301-351	NC_000068.7:c11_1_66050-66214	-0.66	14	17	yes	yes	★★★
